# Mediterranean Aquaculture in a Changing Climate: Temperature Effects on Pathogens and Diseases of Three Farmed Fish Species

**DOI:** 10.3390/pathogens10091205

**Published:** 2021-09-16

**Authors:** Maria Chiara Cascarano, Orestis Stavrakidis-Zachou, Ivona Mladineo, Kim D. Thompson, Nikos Papandroulakis, Pantelis Katharios

**Affiliations:** 1 Hellenic Centre for Marine Research, Institute of Marine Biology, Biotechnology and Aquaculture, 71500 Heraklion, Greece; mariachiaracascarano@gmail.com (M.C.C.); ostavrak@hcmr.gr (O.S.-Z.); npap@hcmr.gr (N.P.); 2 Department of Biology, University of Crete, 71003 Heraklion, Greece; 3 Biology Center of Czech Academy of Sciences, Laboratory of Functional Helminthology, Institute of Parasitology, 370 05 Ceske Budejovice, Czech Republic; ivona.mladineo@paru.cas.cz; 4 Vaccines and Diagnostics, Moredun Research Institute, Pentlands Science Park, Bush Loan, Penicuik, Midlothian EH26 0PZ, UK; Kim.Thompson@moredun.ac.uk

**Keywords:** climate change, fish diseases, Mediterranean Sea, seabass, seabream, meagre

## Abstract

Climate change is expected to have a drastic effect on aquaculture worldwide. As we move forward with the agenda to increase and diversify aquaculture production, rising temperatures will have a progressively relevant impact on fish farming, linked to a multitude of issues associated with fish welfare. Temperature affects the physiology of both fish and pathogens, and has the potential to lead to significant increases in disease outbreaks within aquaculture systems, resulting in severe financial impacts. Significant shifts in future temperature regimes are projected for the Mediterranean Sea. We therefore aim to review and discuss the existing knowledge relating to disease outbreaks in the context of climate change in Mediterranean finfish aquaculture. The objective is to describe the effects of temperature on the physiology of both fish and pathogens, and moreover to list and discuss the principal diseases of the three main fish species farmed in the Mediterranean, namely gilthead seabream (*Sparus aurata*), European seabass (*Dicentrarchus labrax*), and meagre (*Argyrosomus regius*). We will attempt to link the pathology of each disease to a specific temperature range, while discussing potential future disease threats associated with the available climate change trends for the Mediterranean Sea.

## 1. Introduction

Aquaculture in the Mediterranean is of significant economic importance and has exhibited steady growth as well as considerable diversification in the last few decades. It relies mostly on the rearing of shellfish and marine finfish, which account for 98% of production, while freshwater farming occurs to a lesser extent. Finfish farming, in particular, has been growing at a fast pace relative to shellfish farming, and currently accounts for half of the Mediterranean aquaculture production in terms of volume, and over two-thirds in value. The sector focuses on carnivorous species reared in marine cages and is dominated by two species, gilthead seabream (*Sparus aurata*) and European seabass (*Dicentrarchus labrax*) with estimated productions of respectively 200,000 and 208,000 tons of fish per year [1]. These two species make up 95% of total finfish production in the Mediterranean [2]. Greece is the main European producer, accounting for 58.8% of gilthead seabream and 51.1% of European seabass production, followed by countries such as Spain (19.6% and 26.7%) and Italy (7.9% and 9.1%) [3]. Along with Turkey and Egypt, these five countries comprise more than 90% of the total Mediterranean production. In recent years, Mediterranean aquaculture has diversified to include other species that show farming potential, such as meagre (*Argyrosomus regius*), red porgy (*Pagrus pagrus*), sharpsnout seabream (*Diplodus puntazzo)*, and greater amberjack (*Seriola dumerili*). However, while technological and genetic advances have allowed the successful rearing of these species, they still add up to a much smaller percentage of the total aquaculture production in the Mediterranean. The recent increase in meagre production (10,000 tons in 2019 and growing [1]) was the reason for including this species in the review.

Disease outbreaks are the main bottleneck for the aquaculture industry worldwide, being responsible for economic losses on the order of billions of US dollars annually [4,5]. Farmed fish are more susceptible to infectious diseases than wild fish, due to the stressful conditions associated with intensive farming production [6,7]. Infectious diseases in aquaculture also have potential repercussions for the consumers, especially when caused by pathogens that can infect both fish and humans (food-born infections or zoonoses) [8,9,10]. This is also the case for the Mediterranean aquaculture where bacteria as *Photobacterium damselae* subsp. *damselae* or *Mycobacterium marinum*, known for their zoonotic potential [11,12], are not an uncommon finding. To hold off outbreaks and limit losses, common therapies often involve the use of antibiotics. This is the case also for Mediterranean aquaculture, where these compounds are commonly used to treat bacterial diseases [13,14], with serious implications for environmental pollution, antibacterial resistance and the risks of antibiotics residues in fish products [8,15,16,17,18,19].

Anthropogenic climate change is expected to have substantial effects on aquaculture; potential threats for Mediterranean finfish aquaculture, in particular, have long been recognised (reviewed by Rosa et al. [20]). Specifically, significant shifts have been projected for the Mediterranean Sea regarding future temperature regimes, as well as shifts in ocean acidification, currents and water circulation, dissolved oxygen, salinity and the frequency of harmful algal blooms. While each of these shifts will undoubtedly have considerable effects on marine life, it is widely accepted that the greatest impacts will be due to temperature, one of the main drivers of environmental change. Therefore, as we move forward with the agenda to increase and diversify aquaculture production, rising temperatures will have a progressively relevant impact on farmed fish, consequently affecting a multitude of other issues impacting on fish welfare. In fact, elevated temperatures have often been associated with increased outbreaks of diseases that can have severe economic ramifications on the fish farming sector, as well as potential human health-related implications. Thus, increases in water temperature, coupled with high stocking densities typical for intensive fish production systems, such as marine cage aquaculture, may lead to an increased frequency and intensity of disease outbreaks.

Since climate change is already ongoing, it becomes crucial to understand the complexity of its effects on fish health and promptly develop tools and management practices to limit economic damages in the aquaculture industry worldwide. The aim of the present review is to discuss the existing knowledge regarding disease outbreaks in the context of climate change in the Mediterranean Sea and indicate its potential consequences and threats for future aquaculture production. The objective is to generally describe the recognized effects of temperature on the physiology of fish and pathogens, and to specifically list and discuss the principal diseases of common farmed species in the Mediterranean Sea (gilthead seabream, European seabass and meagre). To our knowledge, no studies are available tracking the occurrence of fish diseases in the Mediterranean over time or reporting variations in the frequency of pathologies with respect to rising water temperatures. Undoubtedly, this information would greatly enhance this review. However, due to the clear lack of data describing potential trends in disease outbreaks, an effort was made to collect and compile all the available fragmented information and link each known pathology to a specific temperature range

## 2. Temperature Projections in the Mediterranean Sea

The Mediterranean Sea is broadly divided into the western and eastern basins, which are connected through the relatively shallow Sicilian Channel. The two basins can differ slightly in their temperature profiles, but they generally exhibit mild winters with sea surface temperatures (SST) ranging between 16–20 °C, although in some of the northernmost regions, temperatures may be lower. In summer, typical temperatures range between 24–28 °C, with an important distinction being that the eastern basin generally exhibits higher values of up to 2 °C compared to the western basin [21].

Anthropogenic climate change is expected to cause a significant shift in this temperature range. The global mean surface temperature has already increased by 0.85 °C since preindustrial times, and the most pessimistic assessments predict a further increase of 3.5 °C by the end of the century [22], coupled with an anticipated increase in the frequency of heatwaves mostly across Europe and Asia [23]. The Mediterranean Sea seems to be particularly susceptible to global warming because it is a semi-enclosed basin with low water exchange between other oceanic masses [24]. For reference, oceans have been warming at a rate of 0.11 °C per decade over the last 50 years, while the rate of warming for the Mediterranean has been much higher i.e., 0.61 °C [25,26].

As the magnitude of climate change is predominantly affected by shifts in carbon emissions, trends for future climate change tend to be based on socioeconomic consequences. In its fifth Assessment Report, the Intergovernmental Panel for Climate Change (IPCC) adopted four emission scenarios (Representative Concentration Pathways, RCPs). Of them, RCP4.5 and RCP8.5 are most commonly analysed because they represent the ‘most likely’ and ‘worst-case’ scenarios, respectively [22,27,28,29]. However, the most recent IPCC reports indicate that carbon emissions have already exceeded the threshold defined for RCP4.5, which renders RCP8.5 increasingly relevant [30].

For the Mediterranean, temperature projections for these scenarios are freely available via the EURO-CORDEX initiative (https://www.euro-cordex.net/, accessed on 20 July 2021), which provides high-resolution data downscaled from a wide array of regional climate models (RCM) [31]. These models have been validated by comparing their predictions with historical data-series, such as those obtained via the Copernicus Marine Environment Monitoring Service (CMEMS). Although this allows reasonable accuracy for the open sea and coarse spatial resolutions, climate modelling in the coastal zone is not as robust [32]. Downscaled climate projections are generally incapable of capturing the frequent temperature fluctuations of the coastal regions at a scale that would be highly relevant for aquaculture and tend to underestimate summer maxima whilst overestimating winter minima [33]. Several approaches to correct for this bias exist but require high-quality temperature data at a high spatial resolution. Such data are rarely available [33,34]. In the context of climate change in aquaculture, it is therefore important to consider that temperatures exceeding the projections of RCM may be exhibited at the local scale of a fish farm, especially during heatwaves.

**Figure 1:**
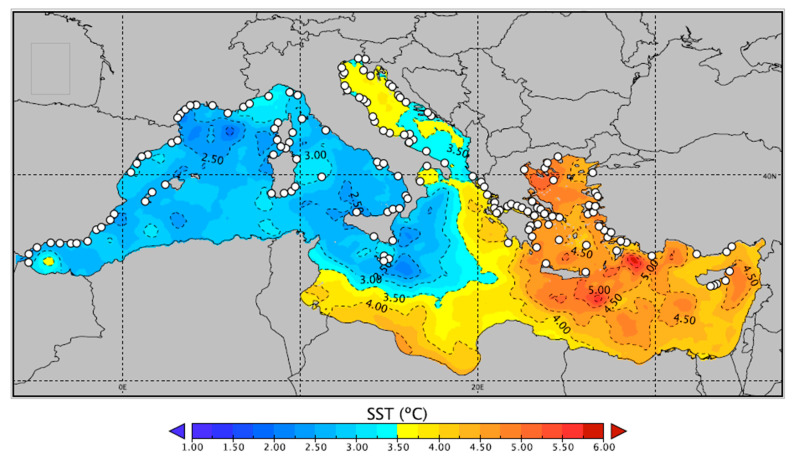
Projection of the average sea surface temperature difference between the year 2015 and 2100 in the Mediterranean Sea. White dots represent the distribution of main known European fish farms. Reproduced with permission from Sakalli 2017 [37]. Fish farm locations were included using data from Med-IAMER (2014) [39].

According to Barredo and coauthors [35] that aggregated and analysed the output of eleven downscaled models, average annual temperatures in the Mediterranean are expected to rise 1 °C by 2050, and 1.9 °C by 2100, under RCP4.5, compared to the reference period of 1981–2010. With respect to the high emission scenario RCP8.5, the respective increases in temperature are projected to be 1.2 and 3.8 °C. For the west part of the Mediterranean basin in particular, such an increase translates to an expected summer SST between 29 and 31 °C by the end of the century, while a maximum SST of 33 °C may also be exhibited during heatwaves, or for the eastern parts of the basin [36]. Other studies have also pointed to a similar increase (Figure 1), thus corroborating the above analysis [37]. In an aggregated output of several regional models and emission scenarios, Adloff et al. [38] predict an increase of 1.7–3 °C for the average Mediterranean temperature, while identifying critical areas such as the Balearic Islands, and the northwest Ionian, Aegean and Levantine Seas, which will exhibit the maximum increase in SST. The increase in temperature seems to be the highest in the summer months (June to August), with RCP8.5 projections being as high as 7 °C by the end of the century compared to current values [22].

It is also important to highlight that the effect of marine heatwaves will substantially exaggerate these temperature trends and cause additional pressure on marine life. These extreme weather events are characterised as temperature anomalies, and, while they have been relatively poorly studied, it is evident that they can have large-scale effects on marine organisms, such as high mortalities and low overall performance that surpass those caused by the inter-annual increase of average temperatures. Admittedly, defining marine heatwaves and setting temperature thresholds for their characterisation is challenging, and while many approaches have been presented [40], this increases the uncertainty regarding future projections of such events. Despite this limitation, Oliver and collaborators [26] reported that, during the last century, marine heatwaves have not only increased in frequency (by 34%) but also in duration (by 17%). This has resulted in an overall increase of 54% of the total heatwave days globally, and although projections at regional levels, such as those relating to the Mediterranean are not available, this trend is expected to continue in the future on a global scale. Specifically, based on data from the last 30 years, Frölicher et al. [41] predicted that, by the end of the century, and depending on the climate scenario considered, marine heatwave days will increase by a factor of 16–23. The latter will result in temperature anomalies exceeding 2.5 °C and lasting over 100 days.

## 3. Host-Pathogen Interplay in the Context of Temperature Variation

**Figure 2:**
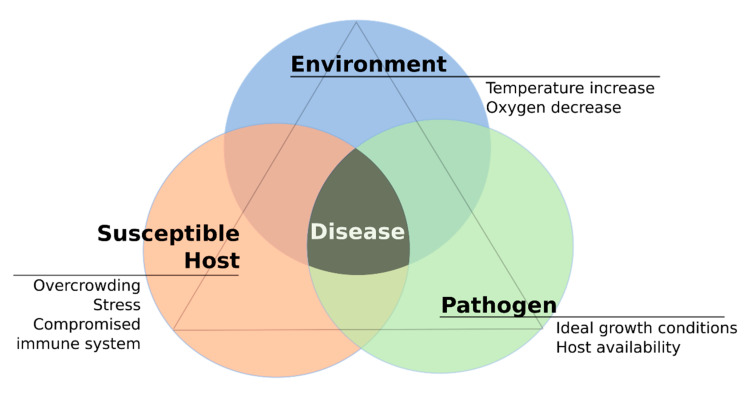
The disease triangle in the context of the increase of water temperature: Interplay of host, pathogen and environment and the main underlying factors that lead to disease development.

It is important to consider that both the fish host and the pathogen are affected by changes in water temperature. Therefore, rising temperatures will be examined in relation to how they affect fish physiology and potential pathogens that consequently can lead to disease outbreaks (Figure 2).

### 3.1. The Host Perspective

Fish physiology is directly linked to different environmental factors such as salinity, light and temperature [42]. Of these, temperature has the most prominent effect (reviewed by Little et al. [43]), to the point that variations in temperature due to climate change are predicted to drive a shift in the geographical distribution of natural populations [44]. Amongst the other environmental stressors that would be altered in the context of climate change, acidification as a consequence of increased dissolved CO_2_ levels has been highlighted as a major obstacle for fish in coping with the acclimation stress [45]. It seems moreover that the response to temperature stress in fish is highly influenced by oxygen limitation; therefore, interplay of multiple stressors is detrimental even when the temperature alteration per se would not impose major pressure [45].

In contrast to indoor facilities, the water temperature of open-air farming systems such as ponds, outdoor tanks and sea cages, cannot be controlled, and is therefore subject to environmental fluctuations. For example, seasonal increase in water temperature is directly related to a decrease in dissolved oxygen levels and has been connected to an increase in stressful conditions within fish farms [46,47].

Temperature changes seasonally during the year and is known to influence embryological development [48,49], growth rates [50], reproductive cycles [51] and immunity [52]. This parameter is, moreover, linked to basal biochemical cellular processes like enzyme activity [53] and structural characteristics of membranes [54]. As we will discuss, the predicted increase in water temperature due to climate change, as well as the increase in prevalence and intensity of extreme weather events, will potentially (1) affect basal metabolic processes, (2) induce a stress response, and (3) differentially impact on various components of the immune system.

#### 3.1.1. Temperature Effect on Fish Physiology

There is a species-specific temperature range within which fish perform optimally, exhibiting high growth rates and robust physiology. Within this optimal range, there is a positive association with temperature, with an increase in temperature typically promoting high metabolic and enzymatic activity and thus assimilation of nutrients and growth [55,56,57]. However, outside this range, biological performance radically declines and once certain critical thresholds are exceeded, vital metabolic processes are eventually suppressed, leading to pathologies and death [58,59].

In fish and in other ectotherms, it has been observed that the limits of the thermal tolerance window of each species are characterized by (1) a progressive switch between aerobic and anaerobic metabolism [44,60] and (2) activation of protection mechanisms, such as expression of heat shock proteins (HSPs) [61,62] and production of antioxidants [63,64,65]. These physiological mechanisms help the fish to cope with the changing environment and, if not above critical thresholds, adapt to the new temperature.

When testing the response of fish to temperature variation, it is necessary to consider that the short-term (acute) response represents 1–120 h time range, while the long-term (chronic) response is 1–4 weeks [66], suggesting that its interpretation must be taken with care when interpreted in the timeframe of decades long climatic changes.

The hallmarks of short-term response are changes in HSPs, being upregulated in the majority of eurythermal fish temperature-dependent transcriptomics experiments [66]. Stenothermal fish that are cold-adapted polar, or warm-adapted tropical species, lack an inducible heat shock response [67]. It seems that, in the latter, the constitutive HSP expression results in denaturation or slow folding of proteins at extremely cold temperatures; therefore, another heat coping mechanisms has been proposed to partake in acclimation [66]. Overall adaptation to short-term temperature shift has a global coordination of stress response combined with the regulation of stress-specific genes dependent on species-specific adaptation [66].

Long-term response however, necessitates adjustment of fish physiology affecting the organism plasticity. Plasticity has been considered here according to Padilla and Adolph [68] as a response characterised by the time lag between the environmental cue and the change in phenotype. In long-term response, HSPs and immunity-related genes are generally upregulated, but at a lesser degree compared to short-term response, while metabolic processes and stress response genes are the most dysregulated. This might be alarming because, if stress response genes become downregulated following acclimation in heat-tolerant fish, the cost of maintenance of homeostasis at higher water temperature will become too high. Consequently, less energy would be left for foraging, growth, and reproduction, eventually endangering fish survival [67].

In addition, fish have the capacity to acquire developmental and transgenerational plasticity. The former refers to the phenomenon when an environmental condition, such as temperature, experienced at an early stage through epigenetic mechanisms can alter the subsequent phenotype, shaping the future plastic response to the specific condition [69]. In contrast, transgenerational plasticity refers to the ability of the offspring phenotype to acclimate to changed temperature by a non-genetic parental influence effectuated across generations [70], helping species that have developed in stable environment to cope with acclimation at the adult stage. In both cases, however, epigenetic mechanisms underlaying the phenomena are unknown.

In the -omics era, identification and quantification of a potential shift from the constitutive response of aquatic organisms exposed to climate change can be elegantly performed at the transcriptional and protein level, even with respect to transcription regulation factors. Oomen and Hutchings [66] reviewed literature focusing on fish transcriptional responses to standard abiotic environmental factors, highlighting those to which fish have evolved a response, but may have been potentially altered through climate changes, therefore altering what would naturally occur. The authors identified 38 fish species whose transcriptional response has been studied in relation to one or more changes relating to temperature, salinity, dissolved oxygen concentration and pH. Even though none of these studies refers to the fish species discussed in this review, transcriptomics of listed abiotic factors still represent a constitutive milieu of fish physiological responses to be used as a baseline model in the coming decades when these abiotic factors are expected to be affected as a result of global climate warming. Each study comprises interaction of specific conditions and organisms, suggesting that the generalist rule “one serves all” cannot be applied. Likewise, plasticity to temperature change can involve a wide range of key processes that can be perturbed in different directions under different experimental conditions or organisms-related traits (e.g., age, size, species, tissue). Based on these studies, authors elucidated that a fish’s ability to evolutionary adapt to environmental change is highly interlinked to the amount of heritable variation that the fish possess for adaptive traits.

Short term experiments have been mostly used to discover temperature tolerance genes that might be of use in aquaculture and breeding, which quickly restore to constitutive levels with no consequence to fitness [71]. These sets of candidate genes seem to vary with respect to species, population, and even at the family level [72,73,74,75,76] and represent the key to understanding the evolutionary machinery behind thermal adaptation in fish. Identifying target genes that facilitate adaptation to temperature might be crucial for developing strategies to face climate change in aquaculture.

While thermal thresholds, especially the ones on the upper end of the temperature tolerance range, constitute knowledge gaps for many of the commonly farmed fish species, empirical evidence suggests that, for most of the Mediterranean species, the survivability threshold does not lie far above the 30 °C mark.

Of the three fish species considered here, seabream is potentially the most susceptible to climate change-induced thermal stress. In natural environments, this fish is known to inhabit the seabed, generally between 10 and 150 m depth, and exhibits winter/summer vertical migrations. According to Feidantsis et al. [77], this vertical distribution is potentially explained as a way for the fish to avoid stress caused by the summer increase in surface temperatures. From their experiments, the authors concluded that temperatures above 20–22 °C induce thermal stress in seabream, as indicated by an increase in the expression of HSP70 and HSP90, and accumulation of lactate (indicating the onset of anaerobic metabolism). Moreover, they observed mortality rates of 5% after 30 days at 26 °C and almost 20% after 10 days in 30 °C, and therefore suggested that the lethal limit for this species is between 24 and 26 °C. If considering that summer surface temperatures in the Mediterranean reach up to 28 °C, while at a depth of 10 m the temperature is usually below 22 °C, their results potentially explain the upper limits of the zonation in wild populations. Additional studies further investigating metabolic and antioxidant patterns and cellular stress markers during natural seasonal variation of temperature already pointed to a potentially poor physiological performance of *S. aurata* in elevated temperature [78,79].

Studies examining the metabolic rate and enzymatic activity in European seabass suggest that the thermal optimum for this species lies between 20 and 25 °C [64,80,81], with increased oxidative stress already evident at 25 °C [65]. While the absolute thermal limits of the species have not been clearly established, recent research with European seabass fingerlings suggests that their overall performance and physiological status deteriorate at higher temperatures, with significant mortalities occurring at 32 °C [82,83].

Interestingly, meagre seems to be the fish species having the highest optimal temperature range among the three fish species examined. This fish is usually reared between 14 to 26 °C [84], and the preferred temperature range for rearing juveniles has been suggested to be between 26 and 30 °C [85]. A study investigating the effects of temperature on meagre, in the range of 20 and 26 °C, showed that most temperature-related stress markers were lowest at 26 °C [86].

Physiological indicators as cortisol levels or osmotic pressure are commonly used to quantify acute stress response to environmental stressors in fish. Interestingly, both of these indicators are known to change seasonally [87,88] and therefore their variation has been directly linked to temperature.

Slightly higher levels of osmotic pressure are observed in warmer periods in meagre, seabream and European seabass [87,88]. On the contrary, cortisol levels display significantly species-specific seasonal differences. A strong positive correlation between cortisol and the annual fluctuation of temperature, with higher levels in warmer seasons when temperature is above 25 °C has been demonstrated in European seabass [89,90]. Conversely, higher cortisol levels are observed in meagre during winter with temperature ranging around 15 °C [88]. In this species, post-stress cortisol levels are statistically higher than baseline levels at higher temperatures (above 25 °C), indicating a lower tolerance to environmental stressors in summer [88].

It is important to remark that all three fish species here considered have consistent differences in both basal (resting) and post stress cortisol levels [91]. Stress-related cortisol levels in seabass are up to 2–4 folds higher than seabream and 20 folds higher than meagre. These variations might be due to differences in basal energetic needs and lifestyles since meagre is considered a sluggish species while European sea bass and seabream are active species with higher energetic needs [88].

Considering that coping with the variation of temperature has an energetic cost on the total energy budget of the organism, species that have lower energetic needs and respond better to stress might adapt more readily to different temperatures.

#### 3.1.2. Temperature Effect on the Fish Immune System

Temperature and stress are two of the main factors known to affect the immune response of fish [92,93,94,95]. As poikilotherms, the metabolism of fish is directly related to their surrounding water temperature, and changes in water temperature are known to affect their immune system. Different fish species have specific immunologically “non-permissive” temperature ranges [52]. As a result, various parameters important to the fish’s immune response can be negatively affected by temperature changes at either end, or outside of their permissive temperature range. This is reflected in the seasonal variations seen in the incidence of disease outbreaks on fish farms as water temperatures change throughout the year.

Various studies have examined the effects of temperature on the innate or adaptive parameters of fish immune systems [52,96,97,98,99] but have tended to focus on the effects of lower environmental temperature on the fish immunity. Less attention has been given to the effects of higher environmental temperature on fish immunity [99,100,101]. It is commonly stated that, in teleost fish, innate immunity is more active at lower temperatures or even temperature-independent [102], while adaptive immunity is suppressed at low temperatures and more active at higher temperatures [96].

Mucosal immunity provides an important first line of defence against invading pathogen [103] whilst remaining tolerant to non-harmful commensal bacteria present in the fish’s microbiota [104]. It would appear that the microbiota of humans can stimulate the development of innate humoral and cellular mucosal responses [105], through the sensing of bacteria signals and metabolites by cells of the innate immune system [106]. The amount of literature relating to microbiota studies in fish is small by comparison, with much of the work relating to gut microbiota in relation to nutritional studies (reviewed by Egerton et al. [107]. There are indications that the developmental stage of the fish, antibiotic treatment, stress, disease and the tank water bacterial composition can influence fish’s microbiota (reviewed by Kelly and Salinas [108]). Limited data are available relating to the effect of temperature on mucosal immunity of gilthead seabream, European seabass and meagre. It has been shown that variations in environmental factors, such as temperature and chronic stress levels, directly affect mucosa and the equilibrium of the microflora associated with it, facilitating the adhesion and invasion of pathogenic strains of bacteria [109,110]. The bactericidal activity of components of the fish mucus has been shown to be compromised after heat treatment using mucus sampled from turbot (*Scophthalmus maximus*), gilthead seabream, and European seabass [109]. As water temperatures increase and pathogens adapt to changing environmental conditions, it is important to understand how thermal stress may compromise the host’s resistance to disease and influence its ability to respond to increased loads/novel opportunistic pathogens present in the aquatic environment [111]. The effect of climate change on the diversity of microbial communities within the fish’s microbiota (and indirect implications for fish health) remains to be established, especially since microbial communities of the fish’s gut seem to become increasingly different to their surrounding environment as the fish ages.

It can be speculated that we will see an increase of skin lesions as a result of increased water temperatures. Intensive aquaculture practices can lead to the formation of skin abrasions, epidermal wounds or damaged mucus layers, resulting in increased accessibility of environmental opportunistic pathogens (favored by high water temperatures) to the tissues and the circulation worsening and expanding skin lesions [112,113]. Skin damage is not only facilitating infection but is also known to lead to life threatening osmotic stress in fish, with as little as 10% damage potentially causing mortality from water loss and ion imbalance [114]. Those that do not succumb have increased metabolic costs from wound healing and osmoregulatory imbalances that may affect their rate of growth and increase their susceptibility to disease [115].

Other mucosal-unrelated innate parameters, such as the alternative complement pathway (ACP) and phagocytosis, have been shown to be affected by temperature fluctuations in the three Mediterranean fish species of interest of this review.

ACP activity is an important component of the fish’s humoral innate immune response involved in pathogen opsonisation and killing. It has been suggested that ACP is well adapted to low temperatures in gilthead seabream [116]. When bactericidal and haemolytic activity (the ability of fish serum to lyse mammalian blood cells as an indicator of ACP activity) was assessed in sera from gilthead seabream exposed to different water temperatures, it was possible to observe haemolytic activity at a very low temperature (i.e., 0.5 °C). Optimum haemolytic activity was observed at 20 °C, however, and this activity progressively decreased at temperatures of 25 °C and above [116].

Phagocytosis is a key first-line defence mechanism of the innate immune system of fish. Respiratory burst, measured as a chemiluminescence (CL) response for the production of reactive oxygen species, was used to determine optimal temperatures for phagocytosis in head kidney cells isolated from European seabass [117]. CL levels were found to be higher at lower temperatures (5–20 °C) than at higher temperatures (25–40 °C). However, in gilthead seabream, low temperatures have been associated with ‘winter syndrome’ [118], a condition related to immunosuppression [119] and activation of a stress response [120]. Prolonged cold temperatures (below 13–15 °C) have been linked to a decrease in lymphocyte, complement, and lysozyme activities in this species, causing low and constant mortalities in farms during winter [121]. Enhanced phagocytic activity has also been observed at lower temperatures for other species such as rainbow trout (*Oncorhynchus mykiss*) [122], and channel catfish (*Ictalurus punctatus*) [123,124], and higher respiratory burst activity noted at lower experimental temperatures [52]. Leukocyte respiratory burst activity and phagocytosis was found to decrease with increasing temperature in carp (*Cyprinus carpio*), while respiratory burst activity increased in rainbow trout and Atlantic cod [125,126].

Studies exploring the relationship between the adaptive immune system and temperature are limited for European seabass, gilthead seabream and meagre, although studies are available for other fish species [127,128,129]. The results suggest that low temperatures suppress primary antibody responses, but that the secondary antibody response can be elicited at low temperature if immunological memory has been established at a high temperature [130,131]. Lower temperature results in a delay in the peak primary antibody response but does not affect the magnitude of the primary response obtained. It has been suggested that the mechanism behind this is governed by one or more thermo-sensitive steps [132], influenced by discrete events during the maturation and/or co-operation of immune competent cells [133,134]. This is not the case for European seabass that seems to display a better response in higher temperatures, with higher antibodies levels observed when fish are held between 24–30 °C, than between 12–18 °C [135].

Unlike mammals, teleost do not show febrile increases in temperature. Instead, fish increase their body temperature by moving to a warmer environment during an infection, a process known as behavioural fever [136,137]. Increase temperature changes during fever and behavioural fever stimulate innate and adaptive immune responses to improve overall disease resistance of the host [136,138,139], and appears to be an evolutionary conserved mechanism between mammals and ectothermic vertebrates to help them deal with infection. The move to an increased thermal preference by fish appears to be triggered through the pathogen recognition mechanisms described above, is controlled by the hypothalamus, and prostaglandin E2 appears to be a major mediator of fever both in mammals and ectothermic vertebrates [137]. What effect increasing water temperature due to climate change will have on this important temperature-related immune mechanism, in both wild and farmed populations, is unclear.

Ultimately, the ability of fish to deal with climate change-related disease episodes will be based on its resistance and tolerance to the pathogen [140]. Resistance to infection relates to a reduction in the pathogen load by the hosts immune system, while disease tolerance is the extent to which tissue damage by the pathogen and immunopathology from immune-driven resistance mechanisms by the host is limited by damage-control mechanisms. There is a fine balance between these two processes to maintain homeostasis in the host, whilst causing the least possible damage to the host’s parenchymal tissues [140]. In a study in which Atlantic cod were experimentally infected with the opportunistic bacteria *Brucella pinnipedialis* at 6 °C (their normal environmental temperature) and a sub-optimal temperature of 15 °C, fish at the higher temperature were able to clear infecting bacteria more rapidly than at 6 °C, and although their immune response was more responsive at the higher temperature, significantly more fish died at 15 °C despite being able to clear the bacteria more efficiently [100]. The authors suggested that the increase in fish deaths might have been due to energy requirements associated with maintaining the physiological homeostasis balance through resistance and tolerance, as well as their growth. They also suggested that there was a trade-off between the cost of immune function and other fitness-related traits like growth, reproduction and thermoregulation, when energy availability was constrained [141]. The balance between disease resistance and tolerance may be harder for the fish to maintain if also having to deal with a thermo-stress response related to climate change.

We can conclude that it is difficult to generalise on what effects climate-associated temperature rises will have on physiology and immune response of gilthead seabream, European seabass and meagre. Each species has different immune components and physiological responses that reacts positively or negatively to changes in temperature (summarized in Table 1). Furthermore, the host immune response is not the only factor to be considered when assessing the effect of temperature on the occurrence of a disease outbreak.

## 4. The Pathogen Perspective

**Figure 3:**
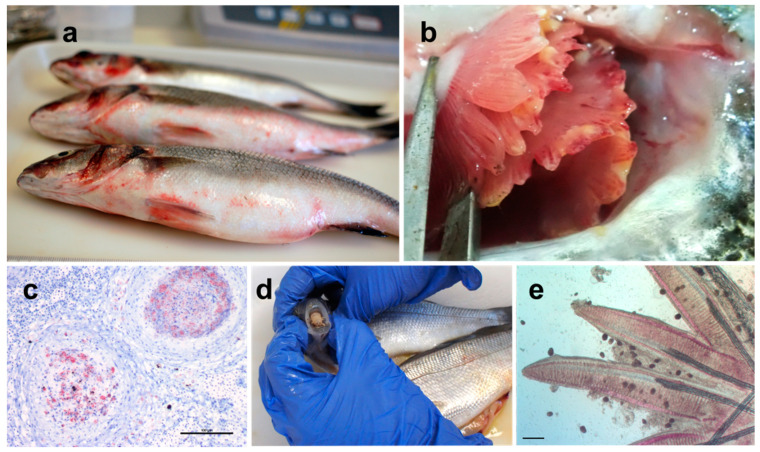
Major diseases observed in Mediterranean finfish aquaculture. (**a**) Vibriosis in European seabass caused by *Vibrio harveyi*. Haemorrhages are visible at the basis of pelvic fins at the operculum and at the anus, common clinical signs of vibriosis; (**b**) Mucoid *Tenacibaculum maritimum* colonies covering the gills of farmed gilthead seabream; (**c**) histological section of European seabass kidney with granulomatous lesions caused by *Mycobacterium* sp. The section was stained with Ziehl-Neelsen, which stains the mycobacteria a vivid magenta; (**d**) *Ceratothoa oestroides* inside the mouth of farmed European seabass; (**e**) fish gills infected with numerous trophonts of *Amyloodinium ocelatum*. Bar: 200 μm.

Several reviews have generically related fish and shellfish pathology to climate change within a global context [20,143,144,145,146,147]. In fact, fish pathogens have been proposed as potential bio-indicators to monitor anthropogenic activity on the environment and climate change [148]. Many different pathogens have been reported as causative agents of fish and shellfish disease in the Mediterranean [149] (Figure 3). Some of these pathogens are strictly host-specific, while others have a wide host range and therefore are ‘shared’ between the three different fish species considered in this review. Several have been identified as pathogens for decades, while others have only recently emerged because of global trading of larval and juvenile stocks or due to transmission from alien/invasive species.

The increase of temperature caused by climate change can moreover favor pathogen migration. Parasites originating from the Red Sea fish are well adapted to warm temperatures and represent a potential threat for native and reared fish in the Mediterranean. One such example, even if unrelated to the hosts discussed in this review, is *Polylabris mamaevi* (Microcotylidae, Monogenea). This Microcotylidae has been described in the Mediterranean [150], although it is naturally associated with the rabbitfish *Siganus rivulatus* inhabiting the Red Sea. Both the parasite and host co-invaded the Mediterranean Sea after the opening of the Suez Canal. The adapted Mediterranean parasite population seems to be more successful compared to the one from the Red Sea, judged by levels of prevalence and intensity being three times greater in the newly colonised region [150].

It should be highlighted that drawing conclusions about the effects of climate change on pathogen–host interactions without analysing both pathogen and host systems together would be insufficient, since the former cannot survive without the latter. This was corroborated when evaluating microbiota inhabiting their host organism under different conditions, as the microbiome can act as a source of opportunistic pathogens depending on the environment. Temperature acclimated oysters, *Crassostrea gigas* (8 or 22 °C), exposed to and surviving temperature stress and *Vibrio* sp. in an experimental challenge, harboured microbiota whose dynamics and bacterial communities were significantly affected by temperature and temperature stress, but not by infection [151]. In contrast, microbiota of dead and moribund oysters expressed community structure disruption, characterised by very low diversity and proliferation of few OTUs (operational taxonomic units). Authors therefore proved the link between microbiota dynamics, alteration of (a)biotic conditions and host survival during disease.

To our knowledge, with the exception of some data on *Photobacterium damselae* subsp. *damselae* [152,153], no Mediterranean aquaculture-related pathogen has been assessed using a molecular approach in relation to temperature changes. A realistic proxy to depict the pathways perturbed by temperature changes is the human pathogen *Klebsiella pneumoniae*; this is an opportunistic pathogen in warm freshwater aquaculture, also propagated through contaminated seafood [154], particularly abundant in natural water reservoirs exposed to temperature variation. It responds to heat shock (50 °C) by generally downregulating gene expression, except for transcripts encompassed within KEGG pathway microbial metabolism in diverse environments. These are included in a wide array of metabolic processes (https://www.genome.jp/kegg-bin/show_pathway?map01120) enabling bacteria to adapt to environmental and metabolic changes, and survive stress conditions in the homeotherm host [155]. Under both normal (20 °C) and heat shock conditions, *K. pneumoniae* upregulates heat shock and ribosomal proteins, suggesting their importance for the cell survival under low- and high-temperature stress.

In conclusion, the same circumstance that shapes molecular responses to temperature changes in fish, listed above, unsurprisingly might be applied to their associated pathogens, although this largely still remains to be corroborated from experimental evidence. For a critical review of a transcriptomic approach to explore physiological responses to the environment, including temperature and lower taxa, readers should consult Evans [156].

Collection of temperature data during disease outbreaks and linking of each pathology to a specific temperature range is the only way of predicting future disease scenarios in Mediterranean aquaculture. However, to our knowledge, no studies have been performed evaluating the association between climate change, predicted temperature trends and the variation in disease outbreak frequencies in Mediterranean aquaculture. Increased incidence of diseases outbreaks is mainly observed by farming stakeholders and diagnostic labs, and this information is often considered commercially sensitive to become publicly available; therefore, it remains unavailable to researchers.

For many of the diseases that are discussed herein, outbreaks have been specifically related to local environmental parameters such as temperature fluctuations or extreme weather events, suggesting a direct relationship between temperature and the incidence of disease outbreaks. In other cases, the information presented has originated from studies conducted to reduce damage resulting from diseases outbreaks by temperature manipulation as part of particular treatment or disease management [150], or obtained from studies conducted in vitro to assess the temperature limits of specific pathogens cultured in the laboratory [157].

Obtaining disease-related temperature information has several associated complications. We observed that many of the published cases report a lack information on temperature or refer to a vague temperature range (either reporting the minimum and maximum annual range or mentioning the month when the outbreak was observed), being mainly focused on disease symptoms and epidemiological traits (e.g., prevalence, intensity and mortality) or merely describing a new pathogen.

To further complicate data collection on associated temperatures, many pathogens have several secondary hosts and complex life cycles, making it difficult if not impossible to retrieve specific information on their optimal temperature range. In some cases, there is conflicting temperature-related data for a specific pathogen between different studies. This can result from the fact that pathogens of the same species can have multiple strains that adapt to a specific environment and co-evolve with their host. The same species can be reported as having different optimal temperatures or different outbreak temperatures if it comes from different areas or different hosts.

Taking all these difficulties into consideration, in most cases, predicting the effect of climate change on a specific pathogen’s fitness and subsequent disease outbreak in Mediterranean fish aquaculture remains mostly speculative.

Four groups of organisms are known to cause pathology in fish: bacteria, parasites, viruses, and fungi (microsporidia). It is beyond the scope of this study to review the extensive literature evidence focusing on molecular mechanisms adopted by each group to cope with temperature shifts, especially when assessed under artificial in vitro conditions. The sole fact that these pathogens are virulent in some hosts and not others directs us towards the role of virulence factors, defined as factors that are essential for microbial replication and survival in a host. For example, fungal virulence factors associated with human infections are associated with its heat-shock proteins, necessary for fungi survival at mammalian temperatures [158].

However, these four groups are still extremely diverse, so we will therefore briefly discuss what is known about the effects of temperature on each one separately. Moreover, because of the difficulty in predicting which pathogens will thrive as a result of changing climate, we have extended our pathogen list (Table 2) also to those that are rarely observed in aquaculture or which induce low levels of mortality in infected fish.

### 4.1. Bacterial Pathogens

**Figure 4:**
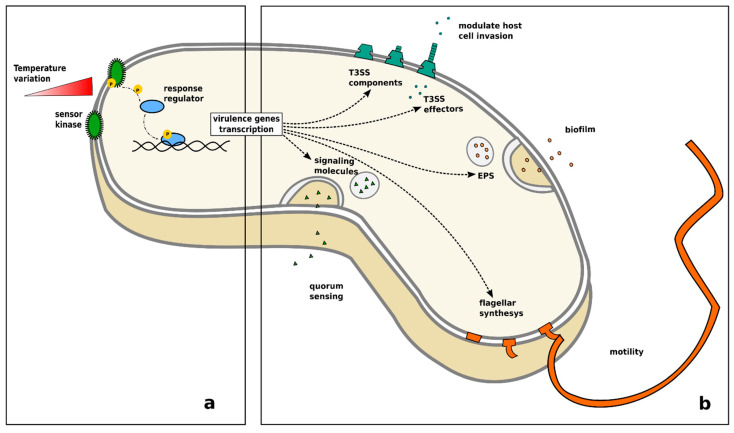
Increase of virulence in bacteria in response to temperature variation: (**a**) change of temperature causes autophosphorylation of a sensor kinase, which in turn activates a cytoplasmatic transcription activator leading to the transcription of virulence genes; (**b**) the release of a quorum sensing signal, biofilm formation, Type III secretion system assembly, synthesis of flagellar components and related flagellar motility have been related to temperature variation in different species of bacteria.

Extensive literature is available on the effects of temperature on bacteria, due to the fact that most bacterial pathogens of fish can be cultivated and studied in the laboratory. Temperature is one of the main environmental factors regulating bacterial life. It is well-known that every species has an optimal temperature range for growth and replication, outside which their growth and survival are dramatically reduced. Bacteria sense environmental temperature changes through a variety of biological thermosensors that act simultaneously on different levels of the transcription chain and on cellular structures (Figure 4a) [255]. Bacterial pathogens are mostly opportunists, meaning their lifestyle encompasses different environments and food sources. Therefore, most species have developed adaptive mechanisms regulated by gene expression to cope with the wide range of environmental conditions they encounter. The shift from a non-pathogenic to a pathogenic lifestyle is directly or indirectly related to temperature via the activation of specialised virulence genes [255,256,257,258] and a number of pathogenic traits, such as the synthesis of flagellar components, motility, the production of a quorum sensing signal, adhesion, and biofilm formation, are known to be temperature-dependent (Figure 4b) [189,259,260,261,262,263,264,265].

An increase in culture temperature can directly enhance bacterial virulence and pathogenicity, as demonstrated in species such as *Shigella*, *Legionella pneumophila* and *Photobacterium damselae* subsp. *damselae* [153,266,267]. This change can be phenotypical (and therefore can be reversed in a scenario of decreased water temperature) [266,267] and genotypical (in a wider evolutionary context) [268,269]. In a study elucidating bacterial evolutionary adaptation to temperature, replicate lines of *Escherichia coli* were propagated for 2000 generations at different temperatures. All groups (even though growing at different rates) showed a progressive improvement in fitness with time (adaptation measure) under their new temperature regime [269]. This improvement in fitness is due to a selection of de novo mutations and is considered as evidence for rapid evolutionary changes in response to temperature shifts [268].

The increase in virulence due to temperature also occurs beyond the farming environment in the open sea, where fish pathogens are less likely to find a host. For instance, the concurrent effect of increasing water temperature and bacteriophage predation on bacterial fitness and pathogenicity in the absence of its host in environmental reservoirs has been studied for *Serratia marcescens* [270]. In a putative scenario in which bacteriophages decrease bacterial growth, resulting in the selection of less pathogenic strains, virulence reduction due to development of phage resistance does not counteract the virulence increase caused by temperature (the phage effect was decreased in magnitude compared to the temperature effect).

It should be noted that not all temperatures can be easily tolerated by bacteria. An increase in temperature may also activate the ‘heat-shock response’ (with a consequent production of HSPs), a well-known mechanism that limits the damage caused by exposure to extreme temperatures [271]. The mechanism of this stress response is associated with impairment of cell viability, abnormal cell morphologies, changes in cell membrane lipid composition and downregulation of virulence factor expression [152].

As a matter of fact, most of the bacteria causing pathology in Mediterranean finfish aquaculture can be cultivated, and it has therefore been possible to establish their optimal temperature range in vitro (Table 3). Other bacteria, like intracellular bacteria in the genus *Mycobacterium* spp. or epitheliocystis agents, are more difficult or impossible to culture and therefore more difficult to study.

#### 4.1.1. Major Bacterial Pathogens

Bacteria of the genus *Vibrio* are responsible for vibriosis, a prevalent systemic disease occurring in most reared fish in the Mediterranean area and worldwide.

*Vibrio anguillarum* is the most studied of *Vibrio* pathogens, being responsible for major economic losses in salmon and eel industries [272,273], as well as in other farmed fish [159]. This pathogen grows rapidly between 25–30 °C [159], with an optimum around 25 °C [191], being characterised by a polar flagellum necessary for the motility, chemotaxis and host invasion [274,275]. The chemotaxis of different *Vibrio* strains towards mucus collected from different surfaces of gilthead seabream (skin, gills and intestinal mucus) has been tested at different temperatures (15, 22, 27 °C) [185]. Chemotactic response towards skin mucus showed to be positively correlated with temperature for both *V. anguillarum* and *V. alginolyticus* and less so but still displayed a temperature-influence towards gill and intestinal mucus. Another study found that chemotactic responses were higher at 25 °C, while swimming speed increased significantly from 25 μm/s at 5 °C to 36 and 40 μm/s at 15 and 25 °C, respectively [192].

The kinetics of adhesion of some *Vibrio* strains on gilthead seabream mucus-coated glass have also been tested at different temperatures [193]. Results were strain- and salinity-dependent and the maximum adhesion was observed at 22 °C for two *V. anguillarum* strains and at 4 °C for two *V. alginolyticus* strains.

*Vibrio harveyi* has been reported as responsible for tail rot disease, causing mass mortalities in larvae and juveniles in farmed gilthead seabream in Malta [276]. It has been found on reared European seabass (Figure 3a) and gilthead seabream in different farms along the Spanish Mediterranean coast [277]. Specifically, different strains of the bacteria have been isolated from the internal organs or ulcers of diseased and healthy gilthead seabream and European seabass, with the latter showing higher overall occurrence. All isolated strains tested for virulence have been demonstrated to be pathogenic in European seabass, while gilthead seabream was apparently unaffected, except for a single strain inducing 10% mortalities. Although the temperature in the study was not specified, authors underlined that the prevalence in both fish species was related to season, occurring only in the warm months between June and November. The difference in susceptibility between European seabass and gilthead seabream has also been confirmed in cases where *V. harveyi* was isolated from the ascitic fluid of gilthead seabream juveniles presenting abdominal swelling [278]. An intraperitoneal injection of the isolated strain in both European seabass and gilthead seabream resulted in lower mortality for the latter. Even though co-habitation trials did not show a horizontal transfer of the pathogen between carriers and healthy fish, asymptomatic gilthead seabream was suggested as a reservoir, representing a threat in areas where both fish species are farmed simultaneously.

*Vibrio alginolyticus* is often associated with epizootic vibriosis in gilthead seabream in the Mediterranean area [186,279,280,281,282,283,284,285]. It was also the most frequently isolated *Vibrio* spp. from the gilthead seabream in a four-year study undertaken in southwestern Spain [281]. As mentioned earlier, temperature-related adhesion is an important virulence factor. This parameter has been established in large yellow croaker (*Pseudosciaena crocea*), where *V. alginolyticus* adhesion on intestinal mucus occurs at 30 °C [286]. Disease outbreaks have been reported in all seasons, as the pathogen can be isolated at 15–25 °C (on TCBS agar), as well as at 37 °C (on *V. alginolyticus* agar) [177]. Frequent mortalities in farmed gilthead seabream in Eilat (Israel) have been associated with this pathogen, both in tanks with temperatures ranging between 22 and 26 °C, and earthen ponds, where higher fluctuations in water temperature occur (i.e., 12–21 °C in winter, 23–33 °C in summer) [186]. In Greece, vibriosis due to *V. alginolyticus* in gilthead seabream is usually observed during the winter months at low temperatures [187], while in Spain, *V. alginolyticus* has been recorded during spring and summer in mixed infections with *V. harveyi*, *V. fischeri* and *V. splendidus* [188]. The synthesis of peritrichous flagella by *V. alginoliticus*, which are responsible for a swarming movement on solid surfaces, depends mainly on temperature and salt concentration [189]. Likewise, studies showed that the pathogen can grow between 20 and 44 °C, but no formation of peritrichous flagella is seen at temperatures above 28 °C or at 0.7% NaCl. In contrast, a higher salt concentration is required for the synthesis of the flagella at temperatures between 30 and 46 °C. The known viable but non-culturable (VBNC) state of this pathogen can be restored with an upshift of temperature (from 4 °C to 26 °C) with or without the presence of nutrients [190].

*Photobacterium damselae* subsp. *piscicida*, formerly known as *Pasteurella piscicida* [287], is a common bacterium responsible for pseudotuberculosis (photobacteriosis or pasteurellosis), a disease characterised by the presence of numerous white nodules (pseudotubercles) on the surface of the internal organs (especially the kidney and spleen) and granulomatous formations [159]. This disease is also related to sudden high mortalities since it can often be asymptomatic. Outbreaks of pasteurellosis are known to occur in summer [159] and have therefore been related to the increase in water temperature in Japan (beginning of summer, 20–25 °C) and in Spain (mid-summer, 25 °C) [288]. The first report of an outbreak of *P. damselae* subsp. *piscicida* in gilthead seabream juveniles occurred in summer in Spain showing 40% of overall mortality [288]. The pathogen was also reported in European seabass along the French Mediterranean coast [289] and in Turkey at lower temperatures (18–19 °C) [174]. In vitro, the bacteria can grow at 15–32 °C, with an optimum at 22.5–30 °C [175]. Asymptomatic gilthead seabream broodstock can transmit the pathogen vertically to larvae that consequently develop the disease once the temperature of water increases. An increase from 15 °C to 18–20 °C increases mortality levels, and a subsequent decrease of temperature (from 20 °C to 15 °C) decreases the mortality, suggesting manipulation of temperature as a disease control measure [176].

*Photobacterium damselae* subsp. *damselae*, initially described as *Vibrio damselae* [290], is an opportunistic pathogen affecting a wide variety of hosts, including humans [11]. Unlike the congeneric *P. damselae* subsp. *piscicida*, most strains of this species can grow at a temperature >30 °C, making this pathogen able to infect homeotherm hosts [11]. Diseases caused by this bacterium in aquaculture have often been related to increased water temperatures during summer months, reflected also from the fact that both bacterial growth and expression levels of virulence factors are higher at 25 °C, than at 15 °C [153]. A recent study investigating transcriptome differences in bacteria grown at 25 and 37 °C suggested that the latter temperature activates a strong stress response that impairs their growth, viability, morphology and virulence [152]. *P. damselae* subsp. *damselae* is a known pathogen of seabream and seabass [291,292,293,294]. Its first isolation was reported from gilthead seabream kept in stressful conditions, under 42 ppt salinity, 26 °C, and overfeeding. Clinical signs included lethargy, haemorrhages at the base of fins and tails, and distended abdomens [294]. A study examining the intraspecific variability of *P. damselae* subsp. *damselae* from different hosts and localities in Spain reported that outbreaks occur mainly in autumn for the gilthead seabream, and in summer for the meagre and European seabass [172]. Moreover, mortality levels in farmed meagre from the southern Spain reached 80% during an August outbreak [173].

*Tenacibaculum maritimum*, formerly known as *Flexibacter maritimus*, is an opportunistic bacterium responsible for “gliding bacterial disease” (or tenacibaculosis) that causes epidermal lesions on the mouth, fins and tail that can become ulcerative, as well as gill (Figure 3 b), and also eye necrosis [182]. The causative agent is a Gram-negative filamentous rod-shaped bacterium with gliding motility. As with many opportunistic pathogens, *T. maritimum* has been reported all around the world in different fish species, being mainly observed when fish stocks experience stressful conditions [182,295]. It can grow in temperatures ranging from 15 to 34 °C, with the optimum being 30 °C [182]. Mortality due to this pathogen in European seabass has been recorded along the French Mediterranean coast and Corsica [183,296]. The latter resulted in 25% cumulative mortality in juveniles, at 12 °C (winter–spring period). Spring and summer, with temperatures >15 °C, have been connected with mortalities along the Aegean coast of Turkey in all European seabass age categories [184]. A collection of eleven strains, with almost identical biochemical profiles, has been isolated from gilthead seabream and European seabass and from several Greek fish farms where this bacterium is extremely problematic [297].

Mycobacteriosis, or piscine tuberculosis, is a worldwide documented disease in fish caused by several species of the genus *Mycobacterium*. It is characterised by chronic and sub-chronic infections of skin and internal organs (mostly spleen, liver and kidney), resulting in nodular lesions and focal granulomas (Figure 3c) [12,159,298]. Aquatic mycobacteria are also known to cause zoonotic diseases, being transmitted mostly from fish to humans (fish tank or swimming pool-granuloma) [12]. The first systemic mycobacteriosis outbreak in European seabass was reported in Eilat (Israel) in 7-year-old farmed fish, reared at 24 ± 2 °C. Isolates plated on Löwenstein–Jensen media at different temperatures (15, 20, 24, 30, 35, 45 °C) exhibited the optimal growth after three weeks at 24 ± 0.5 °C, slower growth at 15 and 20 °C, and no growth at 30 °C [142]. Several events of mycobacteriosis have been reported on European seabass in south Adriatic and Tyrrhenian inshore farms with water temperature ranging between 19 and 21 °C [170]. Pathogen isolation (27 °C on Löwenstein–Jensen) brought to the identification of *M. pseudoshottsii* as responsible for the disease. Another case study described an outbreak in the same temperatures (21 °C in January 2003) along the Aegean coast of Turkey [167]. Farmed meagre are also susceptible to mycobacteria, with an outbreak reported in meagre in Turkey during September 2013. Bacteria were isolated and grown at 24–25 °C [168], while from a summer outbreak in the same year, *Mycobacterium* sp. was isolated and grown at 30 °C [169]. Fish isolates of *M. marinum* display a wide strain variations [299], which likely contributes to observed differences in their temperature requirements.

*Aeromonas* spp. are widely encountered bacterial pathogens in finfish aquaculture, extensively studied because of the severity of their clinical signs leading to high mortalities in infected fish, as well as their wide host range (with cold and warm, marine and fresh water species being affected) [159]. These bacteria have a variety of virulence factors responsible for hemorrhagic septicaemia, cutaneous haemorrhages (mostly on the fins and trunk), exophthalmia, abdominal distension and heavy internal lesions (haemorrhagic catarrh) [159]. Although the optimum temperature for the growth of *A. hydrophila* is around 28 °C, growth can occur up to 37 °C (reviewed by Woo and Bruno [159]), a temperature that allows zoonotic transmission of this pathogens [300]. In aquaculture, outbreaks are usually associated with multiple stressors, including sudden changes of temperature and high temperatures [159]. *Aeromonas veronii* has been reported as an important emerging pathogen of European seabass in Greece [160,301]. This pathogen causes severe mortalities in adult fish when the water temperature is above 21 °C [160]. In the Aegean Sea, *A. veronii* bv. *sobria* is responsible for morbidity at temperatures above 18 °C, thus outbreaks mainly occur during the summer period (June–August) when temperatures range between 24–26 °C [161]. The optimum growth temperature of these bacteria appears to be 30 °C, with no growth obtained below 12 °C [161]. *Aeromonas* spp. infections have been previously reviewed as the principal cause of bacterial diseases of farmed and wild fish in Greece. In particular, a higher occurrence of aeromonads was observed in fry and adults European seabass and gilthead seabream, respectively, with the main *Aeromonas* sp. isolated from these fish being *A. sobria* [302].

#### 4.1.2. Minor Bacterial Pathogens

Epitheliocystis is a disease caused by a variety of intracellular non-cultivable bacteria [303,304]. It is associated with various mortality rates in different farmed species worldwide, mostly affecting larvae and juveniles. The intracellular bacteria replicating inside the host cell form cyst-like inclusions, mostly in gills, causing hyperplasia, respiratory distress, and, occasionally, death. In a recent review, temperature, among other abiotic factors, was suggested as a parameter influencing the prevalence and progression of this disease [303]. Although the inability to isolate and culture the agent in vitro has prevented the determination of optimum growth temperatures, disease seasonality has been observed for different host species in different locations [304]. Farmed European seabass has often been reported as a markedly susceptible species [162,163,305], showing hyper-infection and high levels of mortality, particularly in fingerlings during summer in the northern Mediterranean [162]. Moderate and sporadic infections have also been recorded in South and North France at temperatures 18–20 °C [163]. In addition to seabass, epitheliocystis has been commonly found in gilthead seabream juveniles [3,164,165,166,306]. Although no specific temperature has been given in these studies, the authors state that mild infections in Bardawil Lagoon, Egypt occurred in March, severe infections in the Gulf of Eilat (Israel, Red Sea) in February [164], and acute infections with high mortalities in Spain in the winter of 1994 [165,166]. In Greece, gilthead seabream epitheliocystis was linked to two novel species of β-proteobacteria: Ca. Ichthyocystis hellenicum and Ca. Ichthyocystis sparus [3,307]. These pathogens were responsible for moderate epitheliocystis outbreaks in two different farms in November and high-to-medium infections in other two farms in June and October [3]. Monthly monitoring of the disease during a whole year in another site (Crete, Greece) showed no direct correlation between pathogen prevalence and seasonal fluctuation of local temperature (15–28 °C) (Cascarano personal observation). This, together with the fact that the epitheliocystis is often observed after the transfer of the juveniles from indoor tanks to sea cages, suggests that other factors such as size of the fish and the related stage of development of the immune system are likely to facilitate the occurrence of this pathology.

*Nocardia* spp. are responsible for systemic chronic granulomatous disease in fish, being reported in meagre (Italy and France-imported juveniles) reared in central and western Greece facilities [171,308]. A case study reports growth of *Nocardia* spp. colonies at 25 °C, after a sampling carried out in February 2011, with an annual temperature range of 14–26 °C [171].

*Pseudomonas anguilliseptica* is a Gram-negative motile bacterium with a polar flagellum, responsible for petechial haemorrhages in the skin, mouth, operculum, and ventral part of the body in numerous fish hosts [177]. Cultures of the pathogen grow between 5-30 °C, but not at 37 °C [177]. It has been associated with winter syndrome in gilthead seabream in Spain and France [178,179,180]. In France, outbreaks were observed at temperature below 16 °C, with higher mortalities recorded between 9–13 °C [178]. Mortality rates in different farms in the Iberian Peninsula ranged between 10–15% (with a peak of 30% in some farms) between January (12 °C) until April (18–20 °C) [179,180]. The pathogen motility seems to be temperature-dependent; the number of motile cells increases with the decrease in temperature from 25 to 15 °C in vitro (an isolate from *Anguilla japonica*) [181].

### 4.2. Parasitic Pathogens

Parasites are a broad group of taxonomically-diverged organisms that can be divided into generalists and specialists, according to how selective they are in their choice of host(s). The study of parasitic diseases in marine environments can be challenging since many parasites have evolved complex strategies to inhabit and exploit their hosts. Parasites can have direct or indirect life cycles, the latter involving multiple hosts and consequently undergoing a development that includes different life stages, some of which may be free-living. In most cases, not all these stages are accessible to scientists, and very few models are currently available for the study of marine host–parasite interaction.

It has been observed that increase in temperature can favour a specific life stage of a parasite and consequently its overall success of propagation. This is the case for some monogenean fish parasites (Phylum: Platyhelminthes). Monogeneans include a broad group of lineages of host-specific or generalist, oviparous or viviparous pathogens with a direct life cycle, mostly infecting the external surface of fish, inhabiting specific sites, such as the head, gills and fins, or the extretoro-genital system, where they feed by blood or damage tissue by grazing [309]. It has been proposed that higher temperatures can stimulate the hatching of the eggs and accelerate the life cycle [220,310,311,312]. Moreover, a recent study using the indicator value (IndVal) method that combines measures of fidelity and specificity, while not being affected by changes in abundance, showed that season can influence the occurrence of parasite species in cultured European seabass reared in Corsica, identifying particular species as bioindicators relative to fish farm location [313]. Five parasite species identified as having a significant indicator value for season were the monogenean *Diplectanum aequans*; the copepods *Lernanthropus kroyeri* and *Caligus minimus*; the isopod *Ceratothoa oestroides*, and the myxosporean *Ceratomyxa labracis*. The gill parasite *D. aequans* showed fidelity and specificity more pronounced in winter, in contrast to the copepods and the isopod, which were correlated to elevated water temperatures.

In other cases, specific life stages of parasites are notoriously resistant to extreme temperatures. This is the case for the spores of many myxosporeans. For example, *Myxobolus cerebralis*, a pathogen of salmonids, has been exposed to different temperatures to assess the potential effect of heat as treatment [314]. Surprisingly, its spores have been shown to survive for at least 18 days at −20 °C, remaining unaffected at 40 °C.

Climate change and, generally, temperature fluctuations can directly affect parasite fitness (parasite survival and reproduction) and, moreover, have indirect effects by influencing the hosts on which they depend [315,316,317,318,319].

Ecological models that predict climate change effects on parasitic interactions must consider the influence of temperature on multiple variables. The overlapping of parasite and host thermal preference curves (TPCs), the prevalence, intensity and mode of transmission, and the availability, abundance and distribution of the hosts are all factors that can be positively or negatively affected by temperature [315,320]. Even though each case should be examined independently, it seems that whenever the increase of temperature does not exceed the tolerance limit of the parasite or of the host, the intensity of parasitism and its transmission are commonly favoured (Table 4). This has been shown in a generalised scenario in which the host suffers from oxygen stress and the parasites’ metabolism increases [315].

For parasites with multiple intermediate hosts, this progressive increase in temperature can be beneficial up to the point where it causes mortality in a host, resulting in the disruption of the parasite’s life cycle [321]. Furthermore, very small temperature fluctuations can have significant effects on the dynamics of parasitism, such as in case of fluctuations as low as 0.5–1 °C, making the difference between the exponential growth and the extinction of the parasite [318].

#### 4.2.1. Major Parasitic Pathogens

Infections in farmed meagre by the monogenean parasite *Sciaenocotyle pancerii* (Microcotylidae, Monogenea) have been associated with severe mortality [84,229,230,322]. The parasite was recorded in Sardinia, with the highest prevalence and intensity of outbreaks occurring in September, coinciding with the highest water temperatures, although specific temperature measurements are missing from the study [229]. High mortality was also reported in meagre farmed in Corsica [230]. Here the authors observed the first mortalities in August but started to evaluate prevalence and intensity values from October to January (temperatures ranging between 20 to 14 °C) and observed a peak in November and December. Four monthly samplings were performed to exclude the sampling bias [230]. It is noteworthy that *Sparicotyle chrysophrii*, another microcotylid, shows a general co-occurrence with higher water temperatures in the Mediterranean, except in Corsica as observed for *S. pancerii* [231]. This implies that other biological and physio-chemical traits might cause the proliferation of monogeneans in this geographical region.

*Sparicotyle chrysophrii* (Microcotylidae, Monogenea) (former *Microcotyle chrysophrii*) is a monogenean parasite that infects the gills of gilthead seabream, where it feeds on mucus and epithelial cells, causing tissue damage, leading to anaemia, hypoxia and lethargy [231]. This parasite is responsible for major economic losses in gilthead seabream aquaculture [232,233,235,323]. Reports of outbreaks in reared populations and seasonal studies in different areas of the Mediterranean have shown different trends for this parasite. A study in Corsica recorded the highest abundance *S. chrysophrii* in winter when the water temperature was 13 °C [231]. Similarly, a case report from southern Spain reported increased levels of the parasite at the same water temperatures (13–14 °C) [232]. During the seasonal migration of gilthead seabream from the open sea to a lagoon (spring) and from the lagoon to the sea (autumn) in Southern France, higher abundances were observed in spring (no temperatures mentioned) [324]. Conversely, a study in Alexandria, Egypt, intensity increased dramatically in March (20 °C), maintained high levels throughout the summer (maximum temperature 30 °C), and decreased in autumn and winter (from 20 to 11.8 °C) [233]. From our experience and personal observations in Greek aquaculture, it seems that parasite propagation is mainly influenced by the size and availability of the fish host, with outbreaks occurring throughout the year irrespective of the temperature (Katharios personal observations). The most critical factor is different year classes overlapping within the fish farm, where different age categories are present simultaneously; “donors” (older fish infected with low intensity) and naïve fingerlings recently introduced in cages. Villar-Torres et al. [234] demonstrated that, among different abiotic factors (temperature, salinity, pH and photoperiod), thermal variations caused most perturbation in *S. chrysophrii* infective stages, primarily consisting of reduced development and survival times under higher temperature regime. Authors recorded the optimal thermal range for maximum hatching success to be 14–22 °C, whereas temperatures of 10 and 30 °C likely represent biological thermal limits. In vivo and in vitro experiments have been performed to assess the efficacy of chemical treatments against this parasite. Treatments were tested under temperatures simulating spring–summer occurrence of the parasite, thus adults were kept at 18–20 °C and eggs hatched in two weeks at 20 °C [235]. The hatching took longer when eggs were incubated at 20 °C [235,236] compared to 22 °C [237]. Such acceleration of hatching time and maturation to adult *S. chrysophrii* at higher temperatures could be useful for the treatment schemes applied in aquaculture since most treatments do not affect the eggs.

*Enteromyxum leei* (Enteromyxidae, Myxozoa) is the most important myxosporean parasite causing morbidity and mortality in gilthead seabream, being the most susceptible species of those reviewed herein [149]. The parasite causes intestinal pathology characterised mostly, but not exclusively, by severe enteritis with the detachment of mucosal epithelium. Infected fish develop catarrhal enteritis, displaying anorexia and weight loss, emaciation and cachexia, coupled with poor food conversion rates, and eventual mortality [149,325]. Uncontrollable acute mortalities have been recorded during summer (water temperature of 24–25 °C) in sharpsnout seabream (*Diplodus puntazzo*) farmed in Greece and other Mediterranean countries, which eventually caused the abandoning of sharpsnout seabream aquaculture altogether [211,325]. The most important risk for the transmission and onset of enetromyxosis is water temperature, with optimal development of *E. leei* being achieved at 20–25 °C [209]. In farming conditions, a minimum temperature of 18–22 °C is necessary for development of clinical form in fish [210,211], although, in some farms, disease develops at >20 °C. Enteromyxosis is largely delayed or suppressed below 15 °C, probably because *E. leei* multiplication rate and reaching of an infective dose are limited during winter [212,213]. However, the parasite can remain latent at low temperature, only to reinfect at sudden increase. Therefore, fish that test negative during wintertime can potentially become an *E. leei* reservoir in spring, an important consideration from an epizootiological point of view [326]. Experimental perianal infection conducted at a constant temperature (18 °C), and simulated winter (11–12 °C), autumn (19–22 °C), and summer temperatures (22–25 °C), showed *E. leei* at 100% prevalence at higher temperatures [213]. Additionally, a high prevalence was also observed under constant and autumn temperatures (60–85%), while no infection was reported under winter temperatures. In line with this, Picard-Sánchez et al. [209] observed that one week is enough to infect 100% of fish at high temperature (average 25.6 °C) and 58.3% at low temperature (18 °C). Importantly, high temperatures increased the prevalence of infection in posterior intestine but also supported a higher production of specific antibodies, beneficially limiting the progression of the infection along the intestine.

*Ceratothoa oestroides* (Cymothoidae, Malacostraca) is a generalist parasite in the Mediterranean Sea, common in cage-cultured European seabass and meagre, and to a lesser extent in gilthead seabream [246,247,327,328,329]. This pathogen has a protandrous hermaphroditic life cycle [330]. It starts with infective stages named pulli or manca developing from eggs, infecting the host, settling in its buccal cavity, thereafter losing their swimming capacity (Figure 3d). Through the isopods feeding on blood or by grinding tissues, fish undergo a reduction in growth and weight, showing emaciation and cachexia, but not cessation of eating [331], therefore the most susceptible life stage are fingerlings [246]. An outbreak associated with mortalities occurred through August to November 2000 in a cage farm in Chios (Greece) [246]. The authors highlighted that, during the year of the outbreak, a “prolonged summer” was evident, with temperatures ranging between 21–23 °C. The highest prevalence of this pathogen in Turkish farms in European seabass and gilthead seabream was also recorded during the warmer months [247]. Moving cages to the open sea, where higher currents and lower temperatures could negatively affect parasite development and propagation is helpful although not really a feasible control for the infection. Additional risk for the spread of isopods, indirectly related to an increase in temperature, is the attraction of wild fish to farming sites, especially in warm periods when higher feed loads are distributed within the cages [332]. A recent study showed that *Ceratothoa* efficiently transfers from the wild fish aggregating in the farm site to the farmed European seabass, amplifying the load of infective stages for the latter [333].

*Cryptocaryon irritans* (Holophryidae, Prostomatea) is one of the most common pathogenic ciliates, causing a disease termed “marine white spot disease” or “marine ich” that manifests as skin petechial haemorrhages and skin ulcers in marine fish [334,335]. The parasite is a generalist with a wide geographical distribution, causing massive mortalities, especially in fish farmed in confined spaces, such as inland tanks and aquaria [336,337]. The parasite is common primarily in tropical waters (20–25 °C), with <19 °C not occurring [194,196]. Parasite’s life cycle has been extensively reviewed; it includes a trophont stage (feeding on the host), a tomont stage (leaves the host, encysts and divides into tomites), daughter tomites that later differentiate into infective theronts [334]. As for other parasites, different life stages have different optimal requirements. Cheung et al. [195] studied the effect of different temperatures (7–37 °C) on the reproduction of *C. irritans*. Authors found that encystment of the trophont was maximum at 20–30 °C, while the increase to 37 °C induced 56% trophont mortality and encystment of the rest. The optimal for tomites excystment was 30 °C, while at 37 °C only one cyst showed divisions and tomites did not excyst.

Amyloodiniosis (or velvet disease) is the most common dinoflagellate disease for a variety of fish farmed in warm waters and aquaria [334]. Outbreaks are reported only in the context of aquaculture facilitated by stress and high densities [334], since in natural environments the pathogens has a low prevalence. It spreads rapidly, attaching to the host by a rhizoid root-like structure and damaging the epithelium at the attachment site (mostly gills and skin—Figure 3e) [338]. Inflammation, gill hyperplasia leading to anoxia, haemorrhages and necrosis are common pathological signs, which can often lead to mass mortality events if not diagnosed and treated promptly [339]. Epizootics of *Amyloodinum ocellatum* (Oodiniaceae, Dinophyceae) in gilthead sea bream [338,340], European seabass [338,340] and meagre [341] have been reported. It is an obligate parasite that has three main life stages; a sessile form feeding on the host (trophont), subsequently detaching and becoming a reproductive cyst (tomont) that divides, releasing the motile infective stages (dinospore) [334]. *A. ocellatum* is sensitive to low temperatures, so that no infections occur at <17 °C. Paperna [198] studied the life cycle of the parasite in relation to temperature and provided the optimal temperature for every life stage. Namely, ideal temperatures range between 18–30 °C, gradual mortalities appear at 8 °C and delayed or interrupted division of the tomont occurs at 35 °C.

#### 4.2.2. Minor Parasitic Pathogens

Trichodinids (Trichodinidae, Oligohymenophorea) are widespread ciliates infecting many fish species. They are usually present on the skin and gill surfaces where they feed on mucus and bacteria. In stressful conditions that lead to fish debilitation, they can increase in number, damaging the epithelium by adhesion, crawling and suction [342]. Several *Trichodina* spp. are reported as pathogenic for European seabass and gilthead seabream or other wild fish [163,218,343,344]. Outbreaks in other fish species have been connected with a rise in temperature [345,346]. It has been therefore suggested that temperature might be a major factor favouring *Trichodina* spp. Infections with lower temperatures limiting their proliferation [346].

*Philasterides dicentrarchi* (Philasteridae, Oligohymenophorea), a histophagous ciliate of the subclass Scuticociliatia, has been reported to cause sudden high mortalities in farmed European seabass [347]. The isolate from turbot farmed in North Spain maintained in vitro showed better growth rates at 23 °C than at 18 °C, and no growth at 13 °C, when temperatures representative of yearly fluctuations recorded in the area were tested [347]. Consequently, the optimal temperature range between 18–23 °C was proposed for *P. dicentrarchi*, although temperatures >23 °C were not tested [197].

Ichthyobodiasis (or costiasis) is a common pathology caused by flagellate protozoans from genus *Ichthyobodo* (Kinetoplastea). These pathogens have been long considered generalists with a worldwide distribution in freshwater and seawater [348]. However, it has been recognised that what was considered different parasite strains are actually different species within the *I. necator* complex [349]. These protozoans commonly inhabit healthy fish skin [348], and it is believed that a change in the fish health status or environmental conditions, facilitated by stock crowding, can trigger outbreaks [350]. Pathological infections are characterised by the thickening of the mucus layer, hyperplasia and dullness of the skin, fin and gills lamellar fusion and mortality [350]. The parasite has a free-living motile stage, with a long and a short flagellum, and a pear-shaped parasitic non-motile form (trophozoite) that attaches to the host [351]. *Ichthyobodo* has been described in wild population of European seabass in Portugal [352] and infecting meagre fry in Turkey [353]. High mortality rates (up to 60%) of seabream stocks have been attributed to this pathogen in Turkey [354]. Mortalities of seabream larvae were also reported in intensive open hatcheries in Spain, where fish were kept at 18 °C [199].

Members of the Apicomplexa phylum, such as *Eimeria*, *Goussia* and *Cryptosporidium*, are endoparasitic protozoans responsible for fish coccidiosis [334]. These parasites have a complex life cycle that includes a sexual and an asexual phase, and the infective stages (sporozoites) that mature into coccidian oocysts inside the host cell in different target organs [348]. *Eimeria sparis* (Eimeriidae, Conoidasida) was reported as the most prevalent endoparasite causing trickling mortalities in gilthead seabream in Spain, mainly in spring and autumn [199]. Subsequently, authors described the novel pathogen *E. sparis* and distinguished a novel *Goussia* species (*Goussia sparis*), both co-infecting seabream intestinal epithelium [355]. *E. dicentrarchi* is a poorly known but common coccidian parasite of the intestinal epithelium of European seabass [202,344,356]. In a study conducted in a Croatian farm, an overall parasite prevalence of 16.7% was described on adults and juvenile European seabass sampled in March and October [202]. To our knowledge, no study relating specific temperatures to outbreaks of these parasites has been carried out. *Cryptosporidium molnari* (Cryptosporidiidae, Conoidasida) is another apicomplexan parasite that infects gastrointestinal epithelium through oral transmission via ingestion of oocysts released in faeces. It is isolated from gilthead seabream and, to a lesser extent, European seabass [357]. A study of the mechanism of transmission revealed that infection is initiated at 23.3–26.8 °C [200], and maximum prevalence and intensity in Spain occur in spring and summer [201].

Ichthyophoniasis is a common fish disease with wide geographical distribution and low host-specificity [201] that has been described in Greece in farmed gilthead seabream [358]. It is caused by *Ichthyophonus hoferi* (Ichthyophonidae, Ichthyosporea) that belongs to the Mesomycetozoa phylum, a group of microorganisms phylogenetically close to lineages at the animal-fungus divergence node [359]. It is transmitted through contaminated food, mostly trash or bait fish, but also occasionally through the consumption of small infected fish entering the sea cages, and water. Its proliferation is facilitated by host stress, and ingested *Ichthyophonus* from the intestine enters the blood system and reaches other organs. Its life cycle includes at least four different stages. Thick-walled spores are usually present in the centre of granulomatous tissue of infected organs. This resting stage can germinate into a hypha stage, showing multiple tips (germination tubes). From each tip, a round, thin-walled spherical structure detaches and subsequently subdivides to form a uninucleated motile stage (endospores or infective stage). Interestingly, Spanggaard et al. [360] observed that, in the stomach, the change of pH from 7 to 3.5 triggers the germination of spores into hyphae, suggesting that pH has an important role in the development of the “hypha” stages of this parasite. Spanggaard [203] and Spanggaard and Huss [204], investigating the parameters affecting pathogen’s growth, found no effect of temperature between 0–25 °C, and the lethal temperatures being −20 °C and +40 °C.

*Ceratomyxa* spp. (Ceratomyidae, Myxozoa) are coelozoic myxosporeans that infect the gall bladder of reared European seabass and other wild sparids [211]. They display a seasonal infection pattern, being more prevalent in winter [205]. Katharios et al. [206] reported *C. puntazzi* (described as *C. diplodae*) outbreak in sharpsnout seabream under hormonal treatment for reproduction purposes at 15 °C. In contrast, *C. sparusaurati* from the gilthead seabream gallbladder showed no clear temperature trend, despite the lowest prevalence expressed in summer [207]. Alama-Bermejo et al. [208] developed a first model studying seasonality and infection dynamics in a marine myxozoan, using *C. puntazzi* and wild sentinel fish species. Authors demonstrated that temperature increase induces actinospore production in the benthic invertebrate host, accounting for a double-peaked infection prevalence in fish, i.e., in spring and late summer/autumn (16–24 °C). While no evident infection during the winter months was observed, infective blood stages were present throughout the whole year.

*Kudoa* spp. (Kudoidae, Myxozoa) cause relatively benign infections in the muscle tissue of gilthead seabream, which affect negatively fish post-harvest market value, rather than causing extensive pathology or mortality of the host [342]. *Kudoa dicentrarchi* (former *Sphaerospora dicentrarchi*) and *Sphaerospora testicularis* are myxosporeans affecting European seabass [361,362]. The former is a histozoic parasite that infects connective tissue of the intestine, gall bladder and kidney, and therefore has been transferred into genus *Kudoa*, while the second is coelozoic in testicles’ seminiferous tubules, causing male castration [363]. Infection incidence was observed in the warmer seasons in Spain and Italy [214,215]. The myxosporean parasite *Sphaerospora sparis* (Sphaerosporidae, Myozoa) (former *Polysporoplasma sparis*) is responsible for glomerular disease of the gilthead seabream trunk kidney [216]. The parasite shows a wide distribution in farms throughout the Mediterranean and Spanish South Atlantic coast [364]. Although there is no clear seasonal pattern, prevalence seems to be generally higher in spring and summer [216]. In the Adriatic Sea farms, the parasite was observed at 15–23 °C, peaking during warm months (summer and autumn) [217].

*Diplectanum* spp. (Diplectanidae, Monogenea) are known to cause gill pathology by attachment of their attachment apparatus or opisthaptor that consist of the anchoring disc and marginal hooks or hamuli [365]. Two highly host-specific species have been described: *D. aequans* on the European seabass [218,219,366,367] and *D. scianae* on meagre [368]. Moderate mixed infections of *D. aequans* have been observed at 22 °C in southern Israel [218]. A two-year study undertaken in the Spanish Mediterranean area supported the hypothesis of maximum prevalence and intensity in winter. *D. aequans* “showed a maximal percentage of juveniles in November and peaks in February and May as well. Immature and adult stages exhibited maxima in the months allowing the peaks of juveniles” [219]. Temperatures (retrieved from graph) in February and May were approximately 12–13 °C and 17 °C, respectively, while adults peaked in July at 25 °C. Because of high frequency-infection and eggs’ resistance to pharmaceutical treatments, several studies investigated a *D. aequans* life cycle in relation to temperature [220,221,222]. Testing the effect of six temperatures (from 5 to 3 °C) on egg hatching demonstrated that 83–89% eggs hatched at 15–30 °C, 75% at 10 °C and no hatching was observed at 5 °C [221]. Interestingly, higher temperatures speed up the hatching process, so that the hatching started 2 days following egg deposition at 30 °C, 3 days at 25 °C, 4 days at 20 °C, 7 days at 15 °C, and 11 days at 10 °C. Hatching was completed in 6 days for the first three temperatures, and in 12 and 19 days for the remaining ones. This suggests that *D. aequans* has a wide temperature range, with the optimum at the highest.

*Encotyllabespari* (Capsalidae, Monogenea) is widespread on different hosts, including farmed gilthead seabreams in Egypt, where it showed the highest rates in summer [369].

*Lamellodiscus* spp. (Diplectanidae, Monogenea) are sparids’ parasites with a preference towards larger hosts [224]. In general, they cause limited pathology, and are thus not considered dangerous for farmed fish. Because of their direct life cycle, numerous and fast life-cycle generations, they have been modelled in coevolutionary and host-specificity studies [224]. *L. elegans* has proven capable of switching their sparid hosts in Adriatic farms [370]. The parasite shows the highest prevalence in autumn and spring and the highest abundance in autumn and summer [223,224]. *Lamellodiscus* spp. have also been recorded as secondary pathogens in mixed infections (*Microcotyle* sp., *Trichodina* sp., epitheliocystis and *Vibrio* sp.) of gilthead seabream in winter months in Portugal [225] and northeast Spain [166].

*Furnestinia echeneis*, currently accepted as *Lamellodiscus echeneis* (Diplectanidae, Monogenea) [371], is highly host-specific for gilthead seabream. A comparative study between farmed and wild hosts in three locations showed that the highest prevalence and abundance of occurred in autumn for the farmed fish (average 18–25 °C), while being constant throughout the year in wild fish (lagoon temperatures 15–30 °C) [226]. In Corsica, a heavy infection of *L. echeneis* was observed also in autumn; parasite numbers increased towards the end of summer when water temperatures started to decrease [227]. The authors proposed that summer temperature (23 °C) favours the production and hatching of eggs that develop in adults by the autumn (18–20 °C). The parasite is not considered a significant threat for gilthead seabream, never having been related to mortality.

*Polylabris* sp. (Microcotylidae, Monogenea) are blood-feeding parasites attaching onto gills of perciform fish [372]. *P. tubicirrus* is mainly found in sparids from genus *Diplodus*, causing outbreaks in Corsica (France), while its transfer onto farmed gilthead seabream has been reported in Italy and Greece [373]. It reproduces both in summer (> 25 °C) and winter (10–15 °C) [228].

*Anisakis* spp. (Anisakidae, Nematoda) is a widespread helminth, studied extensively because of its zoonotic character; it is the causative agent of human anisakiasis, a disease of public health concern particularly in the Mediterranean. The prevalence of anisakid infective larvae in wild-caught European seabass (FAO zone 27) ranges between 65 and 85% [374], while no larvae were found in farmed fish [375,376]. The exception is a report in farmed European seabass that observed an accidental prevalence (0.7%) of 2 larvae among 151 fish examined [377]. Anisakids have five life stages, moulting four times and trophically transmitting through at least three different hosts: intermediate and paratenic hosts, such as crustacean, and fish or squids, respectively, and a definitive host (marine mammal) [378,379]. Therefore, it is expected that each stage has different optimal temperature requirements reflected from the host’s optimum. It has been speculated that the egg development and hatching time, and survival of juveniles are species-specific [238], although no recent studies corroborated this. During hatching, the parasite is pelagic (outside the host), which indicates acclimation to offshore temperatures. The hatching percentage and egg hatching time of *Anisakis simplex* were tested under the range of −0.7 to 27 °C. Expectedly, hatching time increased in lower temperatures, while eggs hatched at all temperatures above 1.9 °C [238,239]. Experiments on the survival time of larvae (corresponding to the L1–L3 stage, i.e., when larvae enters the crustacean host) showed that lower temperatures prolonged the parasite’s survival [239], supporting the hypothesis that *A. simplex* is rather adapted to pelagic marine environments with high salinity [240]. Moreover, two of the most important abiotic factors for the potential distribution of *Anisakis* species are mean sea surface temperature and sea surface temperature range, among the rest (i.e., land distance, depth, salinity, primary production) [240]. Interestingly, a recent review on long-term change in the abundance of *Anisakis* spp. and *Pseudoterranova* spp. revealed an increase only in the abundance of the former over a 53-year period (1962–2015). As one of the reasons, authors suggested a long-term climate change that could affect the host susceptibility to infection by compromising the ability of fish to immunologically or behaviorally resist infection, or that facilitates faster growth and shorter generation times in aquatic parasites [380]. Anisakids are eliminated from fish and products for human consumption mostly by thermal treatment. It has been shown that they can survive up to 78 min at 45 °C, progressively becoming susceptible as temperature increase [238]. This can also be indicative of the temperature tolerance limits of the parasite in nature.

*Hysterothylacium aduncum* (Raphidascarididae, Nematoda) is another helminth reported in European seabass [381], which rarely cause human disease [382]. It also has multiple intermediate hosts as anisakids, except that the teleost fish is the definitive host [383]. In wild gilthead seabream from the northeast Mediterranean Sea, a higher prevalence and mean intensity were observed between March and June, suggesting a positive correlation between temperature and *H. aduncum* prevalence [241].

Parasitic crustaceans of the order Copepoda and Isopoda are usually found on the gills, skin, and in fish buccal cavity. They induce occasional post-haemorrhagic anaemia [242,384,385], reduced growth rates and increased mortalities. Their seasonal population increase is well-known in marine farms [386].

*Lerneanthropus kroyeri* (Lernanthropidae, Hexanauplia) is a parasitic copepod that infects the gills of European seabass, causing mechanical damage in the epithelium and secondary bacterial infections [242]. A higher prevalence of the parasite was observed in spring and summer in Corsica, suggesting a preference for higher water temperatures [242]; however, in Turkey, the parasite is present even at lower water temperatures (17.5 °C) [243].

Sea lice of the genus *Caligus* (Caligidae, Hexanauplia) have shown seasonal epizootics in European seabass in the Mediterranean. During a two-year monitoring study in Bardawil lagoon (Mediterranean coast of Egypt), Paperna [244] observed more incidences and higher intensities of *C. minimum* in European seabass during the winter months and early spring, and lower levels in summer and autumn. A maximum number of copepodites and chalimus was recorded in May. Temperatures in the lagoon ranged between 10–16 °C in January and 28–34 °C in July–August [244]. In another Greek lagoon, the prevalence and intensity of four *Caligus* spp. (*C. minimus*, *C. pageti*, *C. mugilis* and *C. apodus)* in European seabass followed the same trend throughout the year, while a high mortality was recorded during winter (8–10 °C) [245]. It was suggested that the higher rates during winter in lagoons can be related to fish migrating for winter basins, where they do not swim vigorously and local currents are minimal, encouraging parasite infection.

### 4.3. Viral and Fungal Pathogens

Very few species of virus and fungi are pathogenic to the fish species reared in the Mediterranean Sea, compared to the number of bacterial and parasitic pathogens that are present. Furthermore, literature addressing the effects of temperature on these viral and fungal pathogens is scarce, and little is known on the potential impact of climate change on their biology.

This gap in the knowledge is especially important for viruses, when considering that these make up the most abundant group of biological entities in the ocean, and their numbers exceed that of prokaryotes by at least one order of magnitude [387]. The majority of marine viruses infect bacteria, archaea and microalgae. The impact of climate change on these has been reviewed elsewhere [388] and is not the topic of this review, but it should be noted that the impact of climate change on bacteriophages may indirectly affect pathogenic bacteria in significant and multiple ways. Bacteriophages are the main drivers of bacterial abundance and the most obvious impact is on their magnitude. In addition, temperature drives the selection between the lysogenic and lytic cycle of bacterial viruses [388]. Lysogeny can shape the fitness of bacteria [387,389] and will impact microbial equilibrium and possibly the antagonism between pathogenic and non-pathogenic strains. Lysogenic conversion may also mediate the transfer of virulence determinants between bacteria [389]. It is evident that these concepts are extremely complicated and very difficult if not impossible to model and predict.

Fish fungal intracellular parasites belonging to the class microsporidia are histozoic pathogens, i.e., known to infect muscles, connective tissue or tissues of other internal organs. They can be directly linked to mortalities, but more often they cause high economic losses related to induced emaciation syndrome or unmarketable fish due to cystic formations and induced deformities. Importantly, the same as myxosporeans, these parasites also exist as spores outside the host. This extremely resistant life cycle stage can persist in the environment for a long time even in extreme conditions until a suitable host is found [390].

#### 4.3.1. Major Viral Pathogens

RNA virus members of the Nodaviridae group are responsible for viral encephalopathy and retinopathy (VER) or viral nervous necrosis (VNN), a neuropathogenic fish disease [342]. These viruses cause lesions in the central nervous system and can infect and cause significant losses in a large number of wild and farmed fish species [391,392]. During infection, the virus can be found within cytoplasmic vacuolations in the retinal, brain and spinal cord cells [393,394,395], resulting in high mortality in both juveniles and adults [342]. Some of the hosts present the clinical signs in contrast to others, such as gilthead seabream [396] and meagre [397], which are asymptomatic viral carriers and reservoirs. One of the first VER outbreaks in the European seabass, the most affected species in the Mediterranean, occurred in summer 1995, in two distinct areas of Greece [251]. Mass mortalities (<60%) were reported in two age classes at 25–27 °C, terminating several months later when the temperature decreased from 23 to 20 °C. Therefore, temperature was reported as an important factor affecting progression of VER. Moreover, the acute clinical form with predominant nervous symptoms was observed at the site with the highest water temperature, while at the site with the lower water temperature, the disease manifested in a subacute form characterised only by external lesions. Two other studies, in sevenband grouper (*Epinephelus septemfasciatus*), redspotted grouper (*E. akaara*) and white trevally, indicated an increase of virulence positively correlated to rise of temperature [398,399]. VER thermal preference was also evidenced in a study determining a method of inactivating the virus that showed that the virus was still infective after a 30-min water bath at 50 °C, and avirulent only at 60 °C [252].

#### 4.3.2. Minor Viral and Fungal Pathogens

Lymphocystis is a chronic tumorogenic benign disease caused by the lymphocystis disease virus (LCDV), a DNA virus infecting the fibroblasts in the interstitial connective tissue [350]. It is common, with worldwide distribution in marine and freshwater fishes [400]. During the progression of the disease, infected cells, mostly on the external surface of skin and fins, become hypertrophic, stop replicating and finally burst, releasing the replicated virus. The disease is usually not fatal, infections resolving naturally in a few days or weeks [250]. Different studies and case reports in gilthead seabream originate from Greece [248,401]. Juveniles of gilthead seabream (20–30 g) with surface cystic aggregations (especially head and fins) showed mortality with seasonal periodicity (unspecified temperature), but healed in 2–3 weeks when kept at 24 °C [248]. Along the Spanish South Atlantic coast, the disease was reported during spring and autumn [249], while Paperna et al. [250] reported outbreaks in November in the Red Sea, with annual temperatures ranging between 23–26 °C. In the bluegill, *Lepomis macrochirus*, the virus replicated successfully between 23–25 °C [402], showing a temperature-dependent incubation (12–15 days at 25 °C, and 37 days at 12.5 °C) [350].

*Glugea* spp. (Glugeidae, Microsporidia) fungi have been described in reared gilthead seabream from the French Mediterranean coast [403]. These microsporidians produced xenomas in the musculature close to the pectoral fins. The viability of spores and the extrusion rate of the filaments, considered as valuable parameters of infectivity, were analysed in a study on spore longevity and resistance to heating and freezing [253]. Neither heating (<30 min at 60 °C) nor freezing (<30 min at −15 °C) could completely kill all spores. Temperature of 40 °C (30 min) did not reduce the viability of fresh spore suspensions, but reduced extrusion rates. Interestingly, a short exposure (3 min) at this temperature caused an apparent activation of the pathogen (extrusion and viability values as high, or even higher than control fresh spores), suggesting that the germination process could be activated thermally.

*Microsporidium aurata* (Microsporidium, Microspora), another microsporidian pathogen, forms large cysts in the peritoneal cavity, connective tissue and intestinal epithelium of gilthead seabreams from the Red Sea [404].

Farmed gilthead seabream can be infected in low prevalence and suffering low, continuous mortality by *Pleistophora* sp. (Glugeidae, Microsporidia). The pathogen is embedding in the gastrointestinal musculature or dorsal body muscles [254,405]. Despite infections being observed at low temperatures, i.e., 10–12 °C (February and March, in Greece) [254], a study using *Anguilla japonica* cell line experimentally infected with *P. anguillarum* showed the completion of the life cycle in 13 days at 25 °C [406], suggesting that the fungi favours higher temperatures.

## 5. Risk Assessment

In the scenario of a rapid increase in seawater temperature (as expected during heatwaves), pathogens, especially viruses and bacteria, have a significant competitive advantage over their vertebrate hosts—and their genomic and metabolic plasticity will allow their faster adaptation to new environmental conditions. For bacteria, this may coincide with increased virulence, potentially leading to more severe epizootic outbreaks. Summer season temperatures will possibly be extended for a longer period. Many of the pathogens discussed in this review result in disease outbreaks coinciding with this season and, under the climate change scenario, their transmission could expand to spring and autumn.

Emerging bacterial diseases caused by pathogens, such as *Aeromonas veronii*, have already begun to pose a significant threat to the European seabass industry in Greece and Turkey (Katharios, personal observations). The recorded epizootiological trends have associated the severity of the disease impacts with high water temperature. We anticipate that new emerging bacterial diseases will take over the “niche” of bacterial infections already managed if not eradicated with the use of commercial vaccines, as in the case of vibriosis caused by *Vibrio anguillarum*. Vibrios, such as *V. harveyi*, may become extremely problematic as the water temperature rises. Novel bacterial pathogens have recently been identified in the area, such as *Edwardsiella anguillarum*, being found to cause problems in reared sharpsnout seabream in Greece [407,408]. Even though this pathogen has not been isolated from the three fish species highlighted in this review, its prevalence in Asian aquaculture, high virulence and the fact that its optimal growth is between 28–30 °C [409] indicate the high likelihood of this pathogen becoming established in a new geographical region. This may have devastating consequences that are hard to predict. Another very important threat is posed by bacteria with zoonotic potential. Although fish bacterial pathogens are rarely zoonotic, since they have adapted to water temperatures below 25 °C, several do have the potential to infect humans, especially immunocompromised individuals. Bacteria like *A. veronii*, *V. alginolyticus*, and *Ph. damselae* subsp. *damselae* have been implicated in severe human infections. These are common pathogens of fish in the Mediterranean. It may only be speculation, but we can expect that a shift to higher temperature optima may be accompanied by the activation of virulence factors implicated in human diseases. Moreover, known zoonotic pathogens, such as *Mycobacterium marinum*, thrive in higher water temperatures and will cause problems not only to fish but the overall image of the aquaculture industry.

Of the known viral diseases that will be positively affected by temperature increases, viral encephalopathy and retinopathy (VER) are the most significant. The causative agent of VER is an RNA virus; these viruses display high mutation rates, and many are prone to regular recombination and reassortment, forming novel genotypes from co-circulating strains. In addition, RNA viruses undergo numerous population bottlenecks as they pass from different hosts and encounter shifts in their selective environment [410]. Although a licensed vaccine is commercially available, possible mutations in antigenic viral proteins and the emergence of new strains with different phenotypes with increased virulence and infectivity could potentially render the vaccine ineffective.

Parasites do not have the metabolic and genomic plasticity of prokaryotes, although their diverse strategies of the evasion and manipulation of the host immune system counter for this. Prokaryotes can be controlled with vaccines, whereas this is not currently possible for parasites. As shown in Table 4, if inside the optimal ranges, higher water temperatures will speed up the life cycle of certain parasites, like the monogeneans. *Sparicotyle chrysophrii* is already the principal threat for farmed gilthead seabream and novel data confirm that their transfer from wild fish to farmed fish is accentuated in warmer seawater (Mladineo, personal observation, H2020 ParaFishControl). The parasite is controlled by regular formalin baths, but this practice is unsustainable, as the chemical is banned in Italy and will likely be banned in the near future throughout the Mediterranean. An acceleration of the life cycle of this monogenean will result in the need for more interventions to control it, which is anticipated to have a significant impact on the sustainability of gilthead seabream aquaculture. *Sciaenocotyle panceri* may create similar issues in meagre aquaculture, although this monogenean is not as widespread as *Sparicotyle chrysophrii* in the gilthead seabream farms, limited also by the number of meagre farming sites. With the expansion of meagre farming and co-farming with the gilthead seabream, it remains to closely monitor whether a transfer of these microcotylid species would be encouraged by an increase in temperature. Namely, *S. chrysophrii*, once considered strictly gilthead seabream-specific, has now been observed parasitising farm-aggregating wild sparids, not solely *S. aurata* (Mladineo, personal observation, H2020 ParaFishControl).

## 6. Future Perspectives

All prediction models agree that the surface seawater temperature of the Mediterranean Sea will continue to rise in the next years [36,37]. It is anticipated that the increase in average temperature will be on the order of 2–5 °C with summer temperatures reaching 29–31 °C by year 2100, while also exceeding 33 °C in particularly vulnerable areas, or during heatwaves. These temperatures are likely to be tolerated by most of the pathogens described in this review, but not by the farmed fish. The impact of raising temperatures is especially concerning for seabream, which shows lower thermal tolerance than European sea bass and meagre, with meagre appearing the most resilient.

The aquaculture industry should be prepared to develop new tools and management practices to mitigate the impact of climate change and, more specifically, the increase of seawater temperature. Interventions can be made in all elements of the disease triangle. However, each has a different degree of implementation and, of course, cost.

From the host perspective, the easier solution is to diversify the production to heat tolerant fish species or gradually increase the production of fish species like meagre, which are more resistant to higher temperature, have a larger thermal window or better respond to thermal stress.

Another possible strategy is selecting breeders for temperature resistant traits. Breeders selection is a common practice in European aquaculture [411]. If the species discussed in this review are going to still be farmed in the following decades, it might become important to evaluate the possibility of selecting breeders not only for their growth performance but also for their plasticity in adapting to different temperatures. Different genotypes respond differently to environmental changes, a mechanism defined as Genotype by Environment interaction (GxE) [412]. This concept has been explored to evaluate environmental sensitivity of aquaculture species (reviewed by Sae-Lim et al. [413]). Genetic improvement toward less temperature-sensitive fish must be further explored in the Mediterranean species.

Further studies on fish hosts should finally focus on species-specific developmental plasticity, in order to clarify the thermal limits in which it is possible to adapt juveniles in the hatchery before exposing them to offshore environmental temperatures.

Control of the environmental parameters can only be achieved if aquaculture is practiced in inland facilities. Recirculating aquaculture systems (RAS) offer full control of the water temperature; however, despite the current trend, the economic feasibility of such ventures is questionable for the time being. On the other hand, cage farming is integrating monitoring tools which allow the collection of reliable and accurate data that would be of great value in the near future for assessing the impact of climate change on both fish and pathogens. Smart aquaculture taking advantage of environmental sensors, internet of things and big data analysis is already becoming a reality for many commercial fish farms.

Investing in fish welfare is possibly the most cost-efficient intervention for mitigating the risks of disease in the context of climate change. Boosting the immune system of fish by providing adequate and high-quality feeds and reducing stress and overcrowding can significantly improve the tolerance of fish for certain disease outbreaks. Finally, investing in the development of new prevention and treatment tools is a prerequisite for the sustainable development of aquaculture. This, of course, is costly and time-consuming since regulatory barriers prevent the prompt and cost-efficient licensing of new products such as vaccines and new antibacterials. This is more prominent in aquaculture than any other food animal-producing sector, due to the fragmentation of the aquaculture industry. The existence of many minor species and in general a small market makes development of such products unattractive for the pharmaceutical companies.

## Figures and Tables

**Table 1:** Tested temperatures for physiological and immune-related parameters in the three examined species. Arrows (↑ & ↓) indicate higher/lower levels if the parameter was challenged in two different temperature ranges.

Fish Host	Tested Parameters	Temperature (°C)	References
gilthead seabream	Thermal stress	≥22	[77]
	mortality	≥30	[77]
	Haemolytic activity (Optimum)	20–25	[116,142]
	Immunosuppression	≤15	[121]
European seabass	metabolic rate/enzimatic activity (Optimum)	20–25	[64,80,81]
	Oxidative stress	≥25	[65]
	mortality	≥32	[82,83]
	Phagocytic activity	↑ 5–20; ↓ 25–40	[117]
	Antibody response	↑ 24–30; ↓ 12–18	[135]
	Increasing cortisol levels	≥25	[89,90]
meagre	Optimum rearing (juveniles)	26–30	[85]
	Increasing cortisol levels	≤15	[88]

**Table 2:** Fish pathogens reported in Mediterranean fish farms. In bold, pathogens related to higher mortalities or frequency of severe epidemics (major pathogens). Fish hosts: 1. gilthead seabream; 2. European seabass; 3. meagre.

Group	Pathogen		Disease Name	Host	Zoonotic	Refererence on Temperature or Seasonality
Bacteria	***Aeromonas* sp.**		Aeromoniasis	1,2		[159]
	***Aeromonas hydrophila***		Aeromoniasis	2	yes	[160]
	***Aeromonas veronii* bv *sobria***		Aeromoniasis	2		[161]
	*Ca*. Ichthyocystis spp.		Epitheliocystis	1,2		[3,162,163,164,165,166]
	***Mycobacterium* spp.**		Mycobacteriosis	2,3	yes	[142,167,168,169,170]
	*Nocardia* spp.		Nocardiosis	3		[171]
	***Photobacterium damselae* subsp. *damselae***			1,2,3	yes	[11,152,153,172,173]
	***Photobacterium damselae* subsp. *piscicida***		Pseudoturbeculosis	1,2		[174,175,176]
	*Pseudomonas anguilliseptica*			1		[177,178,179,180,181]
	***Tenacibaculum maritimum***		Tenacibaculosis (myxobacteriosis)	1,2		[182,183,184]
	***Vibrio alginolyticus***		Vibriosis	1,2		[177,185,186,187,188,189,190]
	***Vibrio anguillarum***		Vibriosis	1,2,3		[159,191,192,193]
	***Vibrio harveyi***		Vibriosis	1,2		
	*Vibrio splendidus*		Vibriosis	1		[188]
Parasites	Ciliate	***Cryptocaryon irritans***	Marine Ich	1,2,3		[194,195,196]
		*Trichodina* spp.	Trochodiniasis	1,2		
		*Philasterides dicentrarchi* (Scuticociliates)		2		[197]
	Dinoflagellate	***Amyloodinium ocelatum***	Velvet disease	1,2,3		[198]
	Flagellate	*Ichthyobodo* sp.	Costiasis	1,2,3		[199]
	Apicomplexa (Myzozoa)	*Cryptosporidium molnari*		1		[200,201]
		*Eimeria* sp.		1,2		[199,202]
		*Goussia* sp.		1		
	Mesomycetozoa (protozoa)	*Ichthyophonus hopheri*	Ichthyophoniasis	1,2		[203,204]
	Myxosporea (Myxozoa)	*Ceratomyxa* spp.		1,2		[205,206,207,208]
		***Enteromyxum leii***	Enteromyxosis	1		[209,210,211,212,213]
		*Kudoa* spp.		1		
		*Kudoa dicentrarchi*		2		[214,215]
		*Sphaerospora testicularis*		2		[214,215]
		*Sphaerospora sparis*		1		[216,217]
	Monogenean	*Diplectanum aequans*		2		[218,219,220,221,222]
		*Diplectanum sciaenae*		3		
		*Encotyllabe spari*		1		[222]
		*Lamellodiscus* spp.		1		[166,223,224,225]
		*Lamellodiscus echeneis*		1		[226,227]
		*Polylabris* sp.		1		[228]
		***Sparicotyle chrysophrii***		3		[229,230]
		***Sparicotyle chrysophrii***	Sparocotylosis	1		[231,232,233,234,235,236,237]
	Nematoda	*Anisakis* sp.		2	yes	[238,239,240]
		*Hysterothylacium* sp.		1,2	yes	[241]
	Crustacea (Copepoda)	*Lernanthropus kroyeri*		2		[242,243]
	Crustacea (Isopoda)	*Caligus*		2		[244,245]
		***Ceratothoa oestroides***		1,2,3		[246,247]
Viruses	Lymphocystis			1,2		[248,249,250]
	**Nodavirus**		Viral Encephalopathy and Retinopathy	1,2		[251,252]
Fungi	Microsporidia	*Glugea* sp.		1		[253]
		*Microsporidum aurata*		1		
		*Pleistophora* sp.		1		[254]

**Table 3:** Known temperature ranges for cultivable pathogenic bacteria affecting Mediterranean finfish aquaculture.

Pathogen	Tested Temperatures (°C)	Min	Optimum	Max	References
*Aeromonas hydropila*		ND	28	37	Reviewed in [159]
*Aeromonas veronii*	4, 12, 22, 30, 37	12	30	ND	[161]
*Mycobacterium* spp.	15, 20, 24, 30, 35, 45	<15	24	30	[142]
*Photobacterium damselae* subsp. *damselae*	15, 25	<15	25		[153]
	25, 37		25	37	[152]
*Photobacterium damselae* subsp. *piscicida*		15	22.5 to 30	32.5	Reviewed in [175]
*Pseudomonas anguilliseptica*		5	5 to 30	<37	Reviewed in [177]
*Tenacibacilum maritimum*		15	30	34	Reviewed in [182]
*Vibrio alginolyticus*		<12	30	37	Reviewed in [177]
*Vibrio anguillarum*	5, 10, 15, 20,25, 30	5	25	>30	[191]

ND: not defined.

**Table 4:** General effects of a gradual general increase of temperature (lowest + to highest ++++) on hosts biology and behaviour, parasites’ life cycle and parasites transmission processes. Adapted from Marcogliese (2008).

Temperature	Effects on Host	Effects on Parasites	Effect on Transmission
+	Altered feeding	Faster embryonic development and hatching	Earlier reproduction in spring
++	Altered behaviour	Faster rates of development and maturation	More generations per year
+++	Weakening of immune defences	Decreased longevity of larvae and adults	Prolonged transmission in the fall
++++	Reduced host resistance	Increased mortality of all stages	Potential transmission year-round
